# Estimation of Fractional Photosynthetically Active Radiation From a Canopy 3D Model; Case Study: Almond Yield Prediction

**DOI:** 10.3389/fpls.2021.715361

**Published:** 2021-08-26

**Authors:** Xin Zhang, Alireza Pourreza, Kyle H. Cheung, German Zuniga-Ramirez, Bruce D. Lampinen, Kenneth A. Shackel

**Affiliations:** ^1^Department of Biological and Agricultural Engineering, University of California, Davis, Davis, CA, United States; ^2^Kearney Agricultural Research and Extension Center, University of California Agriculture and Natural Resources, Parlier, CA, United States; ^3^Department of Plant Sciences, University of California, Davis, Davis, CA, United States

**Keywords:** aerial photogrammetry, canopy light interception, canopy profile feature, digital elevation model, digital surface model, virtual orchard

## Abstract

Canopy-intercepted light, or photosynthetically active radiation, is fundamentally crucial for quantifying crop biomass development and yield potential. Fractional photosynthetically active radiation (PAR) (fPAR) is conventionally obtained by measuring the PAR both below and above the canopy using a mobile lightbar platform to predict the potential yield of nut crops. This study proposed a feasible and low-cost method for accurately estimating the canopy fPAR using aerial photogrammetry-based canopy three-dimensional models. We tested up to eight different varieties in three experimental almond orchards, including California's leading variety of ‘Nonpareil’. To extract various canopy profile features, such as canopy cover and canopy volume index, we developed a complete data collection and processing pipeline called Virtual Orchard (VO) in Python environment. Canopy fPAR estimated by VO throughout the season was compared against midday canopy fPAR measured by a mobile lightbar platform in midseason, achieving a strong correlation (*R*^2^) of 0.96. A low root mean square error (RMSE) of 2% for ‘Nonpareil’. Furthermore, we developed regression models for predicting actual almond yield using both measures, where VO estimation of canopy fPAR, as a stronger indicator, achieved a much better prediction (*R*^2^ = 0.84 and RMSE = 195 lb acre^−1^) than the lightbar (*R*^2^ = 0.70 and RMSE = 266 lb acre^−1^) for ‘Nonpareil’. Eight different new models for estimating potential yield were also developed using temporal analysis from May to August in 2019 by adjusting the ratio between fPAR and dry kernel yield previously found using a lightbar. Finally, we compared the two measures at two different spatial precision levels: per-row and per-block. fPAR estimated by VO at the per-tree level was also assessed. Results showed that VO estimated canopy fPAR performed better at each precision level than lightbar with up to 0.13 higher *R*^2^. The findings in this study serve as a fundamental link between aerial-based canopy fPAR and the actual yield of almonds.

## Introduction

Photosynthesis is transforming sunlight into chemical energy to support the daily activities of most plants and photosynthetic organisms. In this process, solar radiation, as the light source, is used to synthesize carbohydrates from water and carbon dioxide (Barnes, 1893). The spectral range of 400–700 nm is known as photosynthetically active radiation (PAR). PAR can be defined most of the time as the number of photons received per unit area per unit time and recorded as photosynthetic photon flux density (PPFD, μmol·m^−2^·s^−1^) using light quantum sensors. Photon flux density (PFD, μmol·m^−2^·s^−1^) has also been determined using a slightly wider range of spectrum between 300 and 800 nm (McCree, 1972). Furthermore, midday canopy light interception is often defined as the incoming Fractional PAR (fPAR, as a percent) intercepted by a canopy by measuring the PAR both below and above the canopy (McCree, 1966; Lampinen et al., 2012). It indicates actual intercepted PAR (or actual PPFD) that helps growers better understand their orchard productivity and spatial variability (Milne et al., 1992; Rojo et al., 2017).

Canopy-intercepted fPAR is fundamentally useful to reflect the status of canopy biomass development. Particularly in perennial specialty crops, the optimal row and tree spacing need to be designed to maximize light absorption while avoiding overcrowding. In recent years, precision farming has gained more attention to address the issues raised by global population growth, scarcity of farming land, labor shortage, and extreme weather conditions. Advanced orchard management could offer a better solution to minimize cost and maximize profitability. With more accurate fPAR interception data, orchard management could be precisely narrowed down from the orchard level to the block level or even the tree level (Zhang et al., 2016). Each block is an individual unit of the entire orchard, which could be considered when site-specific treatments are available for various block conditions. Some studies have already documented that fPAR correlates with the yield for walnut, macadamia, apple, wheat, and cotton (Robinson and Lakso, 1988; McFadyen et al., 2004; Zhang et al., 2008; Lampinen et al., 2011). Therefore, fPAR could provide critical information in crop yield prediction for growers to manage on-site pre-/post-harvest activities such as fertilization and irrigation control and labor and equipment management. As a leading nut crop in California with more than 2.1 million tons of production in 2019 (USDA, 2020b), almond (*Prunus dulcis*) has an urgent need for early and accurate yield forecasting so that the amount of water and nutrient elements, such as nitrogen, could be appropriately applied under the new regulation of the state to avoid environmental contamination (Zhang et al., 2019). Accurate prediction of almond yield is challenging because of the complex influencing factors, such as environmental temperature, bloom time weather conditions, irrigation volume, soil status, and the crop's genotype and age (Boriss and Brunke, 2005). Among all factors, fPAR is the primary determinant of potential yield in almond orchards (Jackson, 1980; Lampinen et al., 2012). It is worth noting that the estimated potential yield could only be achieved if a particular genotype can produce under optimal orchard management in the absence of biotic and abiotic stress and without experiencing extreme weather during bloom. A recent study has shown that an optimal orchard can potentially produce 58 lb acre^−1^ of dried kernel yield for every 1% of canopy fPAR interception, as measured by a mobile lightbar unit (Jin et al., 2020). Rojo et al. (2017) also indicated that midday canopy fPAR could be used as a fundamental indicator for estimating the maximum potential yield for almond crop when there are no other stressors present. Although midday fPAR describes the potential yield, only a small portion (e.g., ~10%) of the trees may reach maximum productivity (Jin et al., 2020).

Typically, the canopy fPAR interception is measured at the midseason (from June to July for almond and walnut) when the canopy has been fully developed. A previous study has suggested that the midday canopy fPAR measurements could be completed between July and October for mature walnut orchards (Lampinen et al., 2012). However, for immature walnut orchards, fPAR increases throughout the season. Rojo et al. (2017) found a second-degree polynomial curve for midday fPAR interception as a function of time to describe the canopy growth over the growing season in 2012 for almonds, suggesting the need for multiple measurements of canopy fPAR. For this purpose, the fPAR measurement tool should be readily available to use. Besides, it is preferable to estimate the canopy-intercepted fPAR at the early stage of plant growth for yield prediction and nitrogen management. For example, around 80% of nitrogen fertilization is applied to the almond crop by June (Youssefi et al., 2000; Brown, 2020). Therefore, growers could benefit from having sufficient time to respond to excessive or deficient nitrogen supply if the estimation of potential yield can be accurately done before the midseason through measuring the canopy fPAR interception.

Because measuring canopy fPAR interception is important for decision support, developing inexpensive tools would be helpful. Traditionally, fPAR data were collected manually using a handheld device (for example, a commercially available lightbar Ceptometer from METER Group Inc., Pullman, WA) (Grossman and DeJong, 1998; McFadyen et al., 2004). However, it is error-prone and time-consuming because only a limited number of measurements can be taken during a short period at solar noon. Such an approach is impossible to be applied on a large scale. Lampinen et al. (2011) developed a mobile lightbar platform for measuring midday canopy fPAR in almond and walnut. It improved the measurement speed to scan a larger portion of the orchard within ±1 h of solar noon. The mobile unit mainly consists of an array of light sensors mounted on a small field vehicle. While the mobile unit runs through the entire orchard measuring canopy fPAR, a data logger records the measurements at a pre-set sampling rate of 10 Hz with a spatial resolution of 0.28 m at the traveling speed of 10 km h^−1^. Such a mobile platform serves as a useful tool for measuring midday canopy fPAR interception in traditional tree architecture orchards on a relatively large scale that could never be achieved using handheld tools. Although the midday canopy fPAR measurements are not possible for planar tree architecture orchards (i.e., trees are trained to two-dimensional fruiting-wall) using similar platforms, a pipeline of conversion methodologies of tree shadows can be found from Zhang et al. (2015, 2016). Although the mobile platform lightbar has notably advanced and expedited the entire process in orchards, it is still challenging to accurately measure midday canopy-intercepted fPAR as the spatial resolution might vary when the driving speed was not constant (Zhang, 2014). Therefore, some more accurate and affordable means should be investigated, such as using terrestrial or airborne images and three-dimensional (3D) modeling for estimating canopy fPAR interception.

3D model reconstruction is a widely adopted approach to investigate the crown attributes of tree crops for phenotypical mapping [e.g., tree height estimation (Torres-Sánchez et al., 2018)] or agricultural automation researches [e.g., robotic tree pruning (Elfiky et al., 2015; Karkee and Adhikari, 2015)]. Both aerial (Kato et al., 2009; Díaz-Varela et al., 2015; Torres-Sánchez et al., 2018) and terrestrial (Rosell et al., 2009; Underwood et al., 2016; Colaço et al., 2017) methods are used for data collection. Light detection and ranging (LiDAR) and photogrammetric mapping are the two most frequently used and comparable techniques for generating high-resolution 3D models (Filippelli et al., 2019).

LiDAR scanning is one of the most accurate sensing techniques that optically measures the target object's distance by emitting light and receiving reflections. The modeling object is formed by generating millions of point cloud data from the sensor. Kato et al. (2009) developed a visual approach called wrapped surface reconstruction (also known as convex hull approximation) to extract forest tree parameters from airborne LiDAR discrete points. Results showed that the tree parameters, such as tree height and crown volume, can be accurately estimated as long as the individual trees were well-segmented, with up to 0.95 of *R*^2^ when the accuracy was assessed using ground truth data. Furthermore, Underwood et al. (2016) mapped an almond orchard using a LiDAR terrestrial sensing system. They processed point cloud data using the voxel volume approximation method (Henning and Radtke, 2006; Lefsky and McHale, 2008) with each voxel size of 0.001 m^3^ estimates the canopy volume. Eventually, their approach achieved an *R*^2^ of 0.77 between LiDAR estimated canopy foliage volume and actual almond yield using a two-year dataset. Although the LiDAR sensing method always provides highest-resolution 3D modeling for tree crops, it is also expensive and computationally heavy for 3D model reconstruction. The aerial photogrammetry method provides an inexpensive alternative and desired solution.

Torres-Sánchez et al. (2018) employed an airborne photogrammetric point cloud generated by a low-cost red, green, and blue (RGB) sensor to reconstruct the 3D models for almond trees. With the development of the object-based image analysis (OBIA) algorithm, they achieved *R*^2^ of 0.94 and root mean square error (RMSE) of 0.39 between estimated and ground-truthing tree height. When examining the tree area, they achieved *R*^2^ of 0.90–0.94 and RMSE of 1.40–2.14 m^2^ compared with ground-truthing values depending on the flight altitude (50 m or 100 m) (Torres-Sánchez et al., 2015). López-Granados et al. (2019) adopted a similar approach to establish the 3D architecture of almond trees for flowering trait identification and evaluation by assuming the trees are trained in a funnel shape, and the canopies did not overlap with each other. Sometimes, when dealing with super-high-density tree orchards, an entire row volume was estimated as a combined object (Díaz-Varela et al., 2015; Anifantis et al., 2019), where Díaz-Varela et al. (2015) achieved an *R*^2^ of 0.53 when they compared estimated and manually measured olive tree height using airborne photogrammetry method. With such promising accuracies achieved using the aerial photogrammetry method, we, therefore, adopted a low-cost aerial photogrammetry technique for creating the 3D models in this study.

The primary goal of this study was to develop a methodology for estimating the canopy fPAR using aerial imagery and photogrammetry. The goal was to show evidence of a fundamental link between the canopy fPAR estimated by Virtual Orchard (VO) and actual yield. The following are the specific objectives pursued:

1) To develop a complete processing pipeline called VO to accurately measure per-tree canopy profile features;
2) To determine if aerial canopy profile measurements can replace the mobile platform lightbar for estimating the canopy fPAR;
3) To develop predictive models between the estimated fPAR and actual almond yield.

## Materials and Methods

### Experimental Sites

Three experimental almond orchards (Orchards 1, 2, and 3) were selected at the University of California Kearney Agricultural Research and Extension Center (KARE, Parlier, California) for this study. The geographic locations and coordinates of the orchards were shown in Supplementary Figure 1. In total, the three orchards consist of 1,440 trees in 192 blocks and 72 rows. Generally, a block contains 3–17 trees, and a row contains 13–28 trees based on the orchard configurations. For example, Orchard 3 in this study has 21 rows, and each row can be equally divided into four blocks (each block contains three trees), resulting in 84 blocks in total. We summarize the orchard and almond variety information in Table 1. The aerial view of the experimental orchards (including the almond variety information for each row) is shown in Supplementary Figure 2.

**Table 1:** Details of the experimental orchards used in this study.

**Data**	**Orchard 1**	**Orchard 2**	**Orchard 3**	**Total**
Location (latitude and longitude)	N36.599229 W119.515579	N36.598224W119.513250	N36.59980 W119.503137	
Planting pattern	Square	Offset	Square	
Planting spacing	6 × 3 m	6.5 × 4 m	6 × 5.5 m	
Number of trees (sample proportion)	722 (50.14%)	449 (31.18%)	269 (18.68%)	1,440
Number of blocks	83	25	84	192
Number of rows	26	25	21	72
Tree age	6	6	11	
Variety and number of trees (sample proportion)	‘P16.013’^a^ 542 (37.64%)	‘Nonpareil’234 (16.25%)	‘Nonpareil’ 90 (6.25%)	
	‘P13.019’ 104 (7.22%)	‘Wood Colony’108 (7.50%)	‘Butte’ 89 (6.18%)	
	‘Lonestar’ 67 (4.65%)	‘Monterey’107 (7.43%)	‘Carmel’ 90 (6.25%)	

a *There are nine (0.63%) inter-planted pollination trees in ‘P16.013’ rows that were not used in this study*.

### Aerial Data Collection and Preprocessing

For this study, we collected temporal aerial imagery from experimental orchards using an unmanned aerial vehicle (UAV, also known as a drone; Phantom 4 Pro, DJI, Shenzhen, China) that includes an embedded RGB camera. The field of view of the camera's lens was 84°, and the resolution of each image was 5,472 × 3,648 pixels. We collected the aerial images in a grid mission pattern at an altitude of 40 m above ground level (AGL), with an oblique angle of 70° and with a front and side overlap of 87 and 83%, respectively. We could collect imagery in 2 min per acre with such flight mission parameters, and we obtained a ground sampling distance of 1.6 cm per pixel. We preprocessed the images with Pix4Dmapper (Pix4D S.A., Prilly, Switzerland) photogrammetry software to reconstruct a 3D point cloud and generate the digital surface model (DSM) of the orchard. The aerial data collections were conducted four times during the 2019 season on May 28/29, June 26, July 26, and August 7 to investigate the canopy development.

### Canopy Profile Features

**Figure 1:**
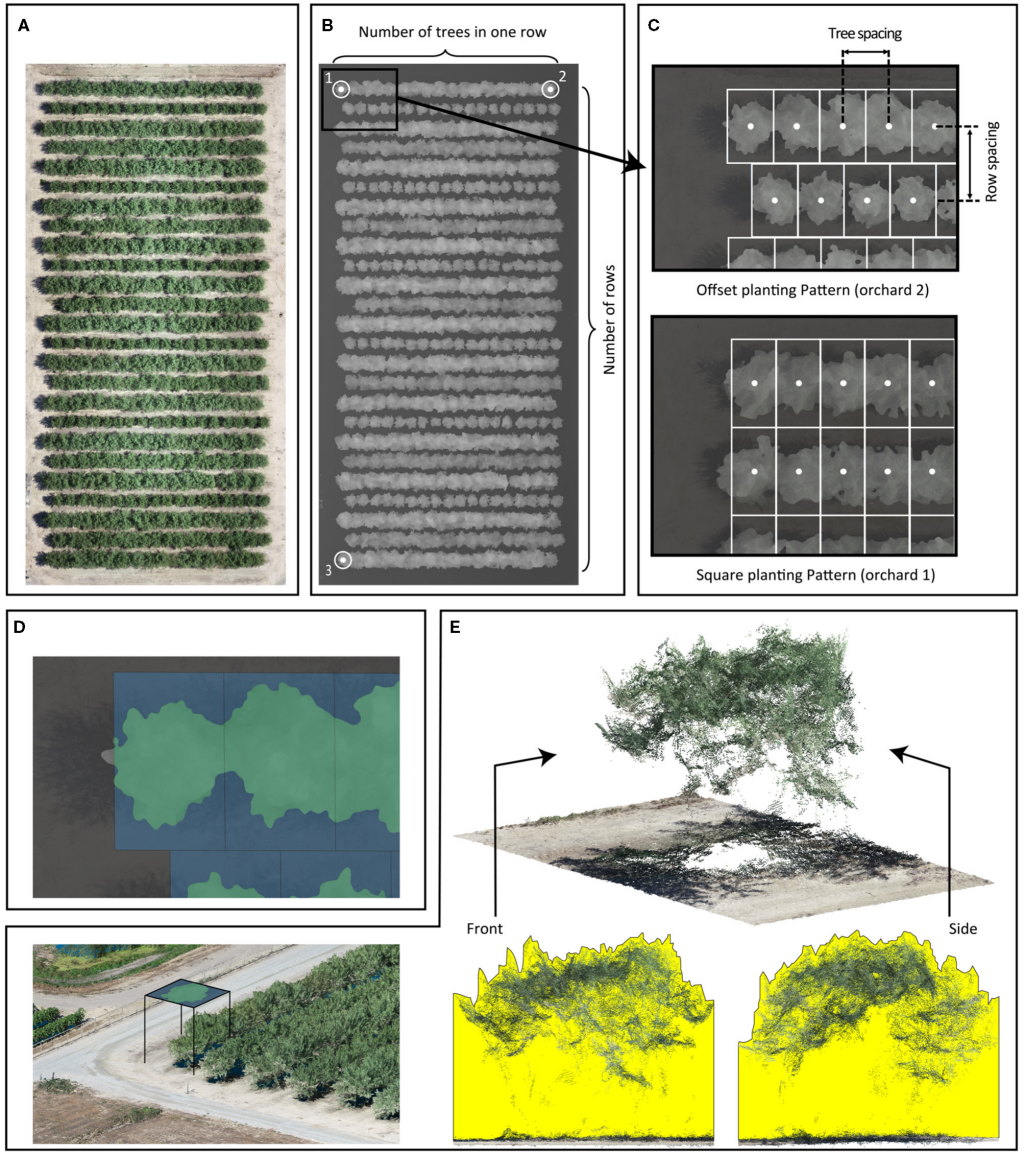
The Virtual Orchard (VO) processing steps: RGB orthomosaic of Orchard 2 **(A)**; a digital surface model (DSM) of Orchard 2 and some input parameters such as number of rows, number of trees per row, and coordinates of tree trunk in three corners: for orchard orientation, three points are placed at the orchard map corners (numeric labels), which form two vectors describing the orientation **(B)**; additional input parameters including row/tree spacing and planting pattern: the offset planting pattern assumes that tree centers in adjacent rows are offset by half of the tree spacing; the square planting pattern assumes that tree centers in adjacent rows are aligned. Tree spacing and row spacing are defined by the distance between the centers of the tree **(C)**; canopy cover is the percentage of canopy footprint area in green divided by the canopy allocated area in blue (Equation 1) **(D)**; 3D point cloud of trees; canopy volume index is visualized in the front and side cross-sectional views of a point cloud generated from photogrammetry for one example tree. The yellow areas represent the front and side cross-sectional views of the volume calculated in the canopy volume index (Equation 2) **(E)**.

We developed a complete processing pipeline, VO library, in Python 3.7 (Van Rossum and Drake, 2009) to calculate canopy profile features. This VO library has been previously used to extract canopy profile features from date palms (Montazar et al., 2020). The tree center points were laid out in a grid by our VO library using user-supplied parameters: the number of trees per row (Figures 1A,B), orchard orientation points (Figure 1B), number of rows (Figure 1B), planting pattern (Figure 1C), and row and tree spacing (Figure 1C). The orchard orientation was defined by three points placed at the tree centers in the corners of the orchard. These three points formed two vectors, where the first vector extends along the direction of the row (formed by points 1 and 2), and the second vector is perpendicular to the first (formed by points 1 and 3). A previous study by Wellington et al. (2012) used a hidden Markov model to determine the tree centers within an orchard, but we could not apply that technique due to the dense planting patterns in the orchards of our study. We calculated the canopy allocated area for each tree using the tree centers, orchard orientation, row spacing, and tree spacing.

To extract the canopy profile features, we segmented the trees from the ground in the DSM raster using a two-step process. First, we created a digital elevation model from the DSM to normalize tree heights from above sea level to AGL. DSM pixels belonging to trees were first identified by calculating the slope using the “richdem” Python library (Barnes, 2016). DSM pixels with a slope >20° (an empirically determined threshold) were classified as trees in a binary mask. A closing morphological operation using a circular, 50-pixel diameter structural element was then applied to the binary mask to fill in the holes where the canopy slope was <20°. This step created a closed binary mask. We selected the structural element's size based on the size of the holes and the resolution of the DSM. We then inverted the closed binary mask and applied the inverted mask to the DSM to segment ground pixels. The missing ground pixels (under the tree canopy in DSM) were predicted using a nearest-neighbor interpolation technique. Second, we extracted the canopy area by segmenting pixels with a minimum elevation of 0.5 m above the ground in the normalized DSM. We used the contour approximation method from the OpenCV library (Bradski, 2000) on the segmented DSM to create a vectorized polygon that overlaid each tree's canopy footprint (Figure 1D). For tree canopies that extended beyond its canopy allocated area, the canopy footprint polygon was clipped to the allocated area's extent. We collated the polygons into a shapefile and used it for further canopy feature extraction.

We extracted four canopy profile features from the normalized DSM, including canopy cover, canopy volume index, average canopy height, and maximum canopy height. Canopy cover was calculated as the percentage of the canopy allocated area filled by the canopy footprint area (Equation 1 and Figure 1D) and used as an estimation of fPAR by VO method (Montazar et al., 2020). More specifically, the canopy allocated area for each tree in an orchard was identical (as the denominator); the larger canopy footprint area (as the numerator) hypothetically represented the more intercepted light by the canopy (fPAR). No physical holes were found in any of the almond tree crowns during data processing in this study. The canopy volume index (Figure 1E) was calculated by taking the ground adjusted height values within each tree's canopy and multiplying it by the pixel area (i.e., defined by spatial resolution of the DSM). The canopy volume index included the volume between the tree crown surface and the ground by summing up the volume of each pixel (px) as calculated with Equation (2), where N is the total number of tree pixels; *Area*_*px*_ is defined by the DSM's spatial resolution. The average and maximum canopy heights were also calculated for each tree but not directly used in this study. All four extracted canopy profile features derived from DSMs (including fPAR, volume index, average height, and maximum height) were visualized at the per-tree level for each orchard from May to August in Supplementary Figures 3–5.

(1) $$\begin{array}{l} Canopy\;cover\;=\;\frac{Canopy\;footprint\;area}{Canopy\;allocated\;area}\times 100\% \end{array}$$

(2) $$\begin{array}{l} Canopy\;volume\;index\;=\;\sum_{1}^{N}Area_{px}\times Height_{px} \end{array}$$

### Ground Truthing

Two types of ground truth data were collected in this experiment, including yield information and mid-day canopy light interception. We harvested each tree separately and measured the weight of wet yield that included nuts and debris, such as leaves and sticks. A tarp was placed at each side of the tree to catch the crops as a mechanical shaker shook it. The nuts and debris caught by the tarp were placed in plastic bins and weighed. We collected and dried subsamples for every block in each orchard. For example, there were 8–17 trees per block in Orchard 1, depending on the orchard configuration. We then separated and cracked the nuts and measured the dry kernel weights per subsample. We established a wet yield to dry kernel yield correlation to estimate dry yield for each tree. Finally, the dry kernel yield per acre was measured for each tree by normalizing dry kernel weight by canopy allocated area.

Canopy light interception, or fPAR, was collected using a mobile lightbar platform (Lampinen et al., 2011). The lightbar platform mainly consists of an array of light sensors (generally with 18 ceptometer segments; Lightbar Ceptometer, METER Group Inc., Pullman, WA) mounted on a small field vehicle. During solar noon (±1 h), the field vehicle scanned the entire orchard to obtain the canopy midday fPAR by computing from PAR recorded below the canopy (*PAR*_*below*_) at the height of 0.4 m and above the canopy (*PAR*_*above*_) simultaneously (Equation 3). A data logger recorded the measurements at a pre-set sampling rate of 10 Hz with a spatial resolution of 0.28 m at the traveling speed of 10–11 km h^−1^ (Zarate-Valdez et al., 2015). A differential Global Positioning System and a radar system recorded the mobile platform's geospatial data during the data collection (Zarate-Valdez et al., 2012). This type of mobile unit is widely used for mapping nut crops in California (Lampinen et al., 2012). We scanned all three almond orchards using such a mobile lightbar platform on June 20, 26, and 27 in 2019. The lightbar estimated midday canopy fPAR data were only available on the per-row/block basis due to its data processing constraints.

(3) $$\begin{array}{l} fPAR_{measured}=1-\frac{PAR_{below}}{PAR_{above}} \end{array}$$

### Statistical Analysis

**Figure 2:**
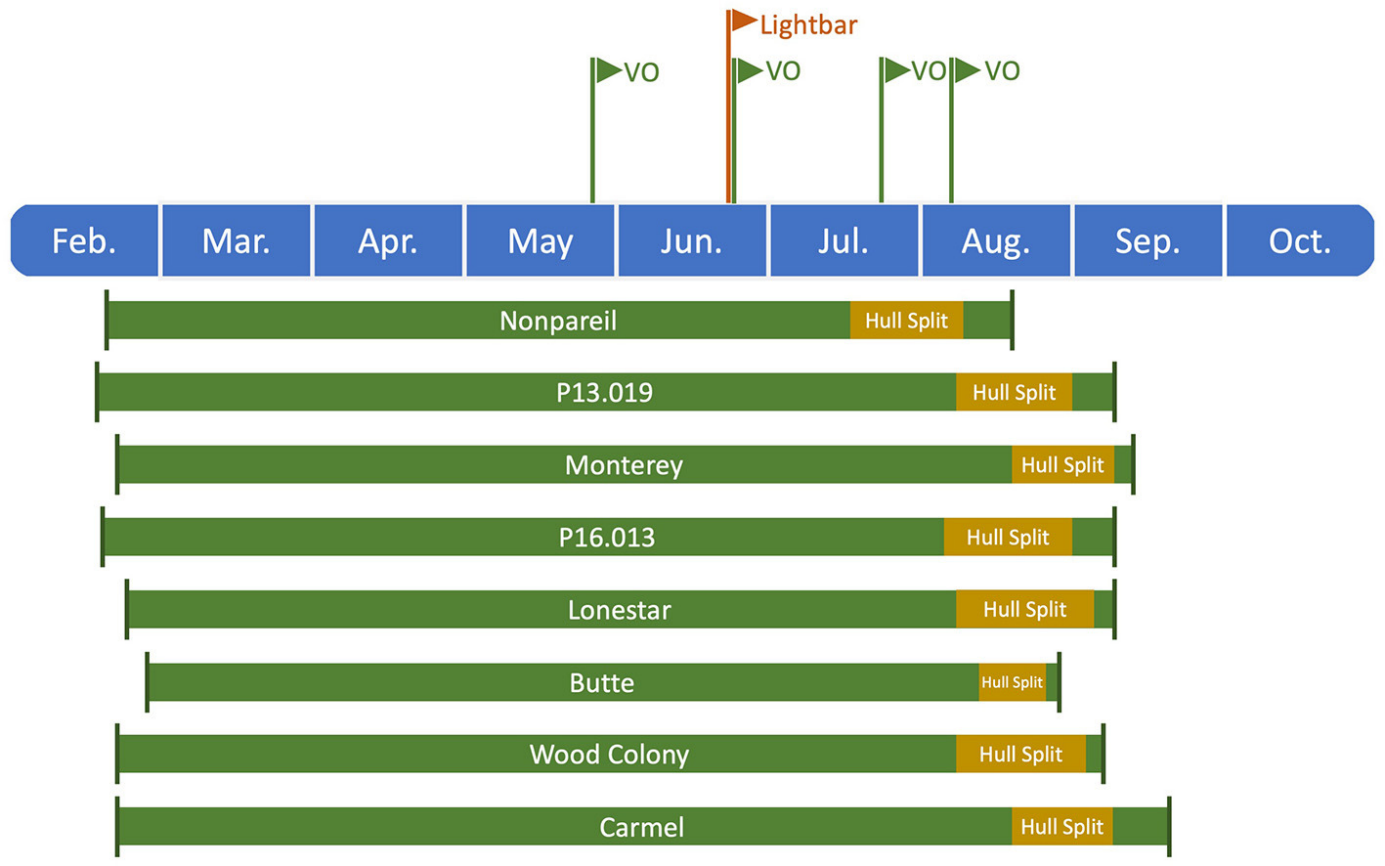
Timeline of the growing season for different almond varieties from full bloom to commercial harvest dates in 2019. All harvest dates were recorded from the experimental orchards, while other dates (i.e., full bloom and hull split) were estimated based on the studies of Connell et al. (2010, 2017) and Lampinen et al. (2017), except full bloom date for ‘Nonpareil’ was also recorded from the experimental orchards.

We conducted statistical analysis to determine the correlation between aerial and ground truth data. In a temporal analysis, we compared the fPAR estimated by VO from different dates in 2019 with fPAR measured by the mobile lightbar platform in June. The VO data collected on May 28 and 29 is abbreviated as May 28, and lightbar data collected on June 20, 26, and 27 is abbreviated as June 26 in the rest of the paper. Figure 2 visualizes the overall growing season timeline (from full bloom to California's commercial harvest dates in 2019) for all almond varieties used in this study. Harvest date referred to the date when trees were shaken or the date of the first round of shake if multiple shakes were applied to the orchard. The nuts were left on the orchard floor for 3–11 days before being picked up.

Although we collected data for eight different almond varieties (i.e., ‘Nonpareil’, ‘P16.013’, ‘Butte’, ‘Carmel’, ‘Lonestar’, ‘Monterey’, ‘P13.019’, and ‘Wood Colony’), we separately analyzed the data for ‘Nonpareil’, which is the leading variety in California (~43% of almond acreage is ‘Nonpareil’) (USDA, 2020a). Overall, ~23% of almond trees are ‘Nonpareil’ in our study. For statistical analysis, we used Python for generating regression models, calculating the standard measures of R squared value (*R*^2^), root mean square error (RMSE), and Pearson's correlation coefficient (r). Besides, we also calculated predictive *R*^2^ (to determine how well the model predicts a removed data point) using SAS software (University Edition, SAS Institute, Cary, NC) in Equation (4):

(4) $$\begin{array}{l} predictive\;R^{2}=1-\left(\frac{PRESS}{SST}\right) \end{array}$$

where PRESS represents the predicted residual sums of squares, and SST represents sums of squares total^1^ Statistical significance tests were performed using analysis of variance (ANOVA) multi-comparison (p < 0.05) and/or Tukey's honestly significant difference (*p* < 0.05). We compared the actual yield to the potential yield (*Y*_*potential*_) calculated by Equation (5) based on a previous study (Jin et al., 2020). They concluded that there are about 57.9 lb acre^−1^ (ratio = 57.9) of dried kernel increment with every 1% of midday incoming fPAR intercepted by the canopy (*fPAR*_*measured*_) in midseason for all varieties; while this ratio changes to 57.7 lb acre^−1^ for ‘Nonpareil’ only. Finally, we employed the Python to visualize the results.

(5) $$\begin{array}{l} Y_{potential}=ratio\times fPAR_{measured} \end{array}$$

Canopy light interception, or fPAR, is the determinant for potential (maximum) almond yield (Lampinen et al., 2012). The trees may reach the potential yield with ideal environmental and internal factors during the production process, and midday fPAR should provide sufficient information about the potential yield when no other stressors are present. In reality, the actual yield is most likely far behind the potential yield due to stresses caused by unavoidable events or non-optimal orchard management. To further quantify the gap between the potential and actual yield from different dates, we developed date-specific models based upon two ratios provided in Equation 5. Consequently, eight *ratio*_*adjusted*_ (or models; from May to August for all varieties and ‘Nonpareil’) are presented using Equation 6, where the slopes between the two measures are calculated by forcing a zero intercept against the 1:1 line.

(6) $$\begin{array}{l} ratio_{adjusted}=ratio\times slope \end{array}$$

## Results and Discussion

### fPAR Estimation

#### Canopy Feature Selection

Some previous studies investigated the correlation between estimated midday canopy fPAR and almond yield with mobile lightbar scanned data. For example, Jin et al. (2020) employed a 10-year canopy light interception (fPAR) data in California, showing a 0.60 of Pearson's r coefficient with the actual almond yield at the orchard level. Obtaining instant and more precise orchard information can help growers to manage orchards at a smaller scale, such as at the per-row, per-block, or even per-tree level, and to promptly respond to deficiency of nutrition elements (e.g., nitrogen) or irrigation volume at a site-specific manner during the critical stages (Brown, 2020). Zarate-Valdez et al. (2015) have developed a methodology with imaging the canopy shadows from a ground vehicle and achieved a good correlation with lightbar data (*R*^2^ was up to 0.95) in almond and walnut orchards. We did not find relevant publications using aerial imagery-based technology that is more convenient and provides much higher spatial resolution (0.01–0.02 m) than a typical mobile lightbar system does (~0.4 m).

We created canopy cover (Equation 1) and canopy volume index (Equation 2) as the two main UAV-based VO canopy profile features. To determine which one is more correlated to the mobile platform lightbar data for estimating fPAR, we compared the per-row canopy cover and canopy volume index. Overall, we found that VO canopy cover is much better correlated to lightbar data than the canopy volume index. Table 2 shows the correlations comparing between both features to lightbar estimated fPAR in June. The Pearson's correlation coefficient (r) is 40 points better for the VO canopy cover. This result is interesting because, theoretically, lightbar estimated fPAR contains spatial information by considering the solar Zenith angle. Rojo et al. (2020) showed a digitized image of fPAR intercepted by almond trees scanned by a lightbar platform, where the darker pixels represent greater light interception than gray-white pixels. The color gets lighter from the tree center to the edge (from the top view) due to the canopy density decrease. This observation indicates that the canopy volume index, which automatically takes the canopy height into account (calculated using Equation 2), should better correlate to the lightbar data. However, we achieved a satisfactory RMSE of 2.94% (to the 1:1 line) comparing the canopy cover and the lightbar estimated midday fPAR, while the canopy cover feature entirely ignored the solar Zenith angle. It indicates that the VO canopy cover feature should contain sufficient information that we need to understand the amount of light intercepted by the canopy, but we need to further confirm this by predicting actual almond yield. Therefore, we selected the profile feature of canopy cover as the VO estimated fPAR hereafter to compare with the mobile lightbar estimated midday fPAR in the rest of the paper.

**Table 2:** Correlation comparison between Virtual Orchard (VO) features of canopy cover, and canopy volume index to mobile lightbar platform (both on June 26) estimated fractional PAR (fPAR) at the per-row level.

**VO profile feature**	***R*^**2**^**	**RMSE^**a**^ (%)**	**Pearson's r**
Canopy cover	0.91	2.94	0.95
Canopy volume index	0.30	12.98	0.55

a *RMSE refers to the root mean square error to the 1:1 line*.

#### VO Temporal Analysis

**Figure 3:**
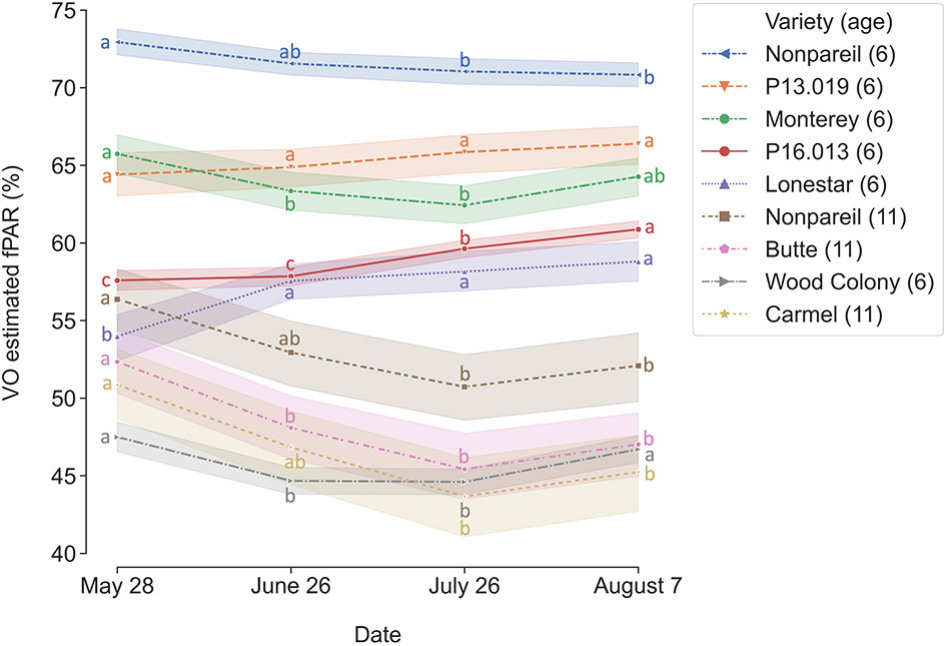
Changes of canopy fractional PAR (fPAR) estimated by Virtual Orchard (VO) over the growing season in 2019. Different letters refer to a statistically significant difference over the season for each variety and age using Tukey honestly significant difference (HSD) test (*p* < 0.05). The size of the confidence intervals of 95% was drawn. Means ± standard deviations (SD) of the yield (lb acre^−1^) for each group were 2000.76 ± 385.38, nan (no data available), 2121.86 ± 572.39, 1013.60 ± 395.84, 1558.49 ± 520.28, 1055.28 ± 386.69, 988.85 ± 463.91, 1436.00 ± 250.07, nan, respectively, from the top to the bottom of the legend.

To determine how tree canopy changes over the season, we conducted a temporal comparison of VO estimated fPAR for different varieties. Figure 3 shows the overall trend of VO estimated fPAR with individual almond variety and age over the growing season in 2019. In general, these different changing patterns might directly relate to the status of individual orchard management and practices. For example, canopy changes were similar for the trees in Orchards 2 and 3 that VO estimated fPAR first dropped a little from May to June or July and then bounced back in August, showing a “flat-U” shape for most of the trees excluding 6-year-old ‘Nonpareil’ trees continuously decreased during the season. All 11-year-old ‘Nonpareil’, ‘Butte’, and ‘Carmel’ varieties are from Orchard 3, where the trees might be under-irrigated during the midseason (June and July), causing a certain level of defoliation. Lampinen et al. (2012) reported continuous increases in lightbar estimated midday fPAR over time for both young (3-year-old) and mature (10-year-old) walnut orchards between April and November 2010. However, Rubke (2015) found that midday canopy fPAR increased only during the 2012 growing season, with data collected over 3 years (2012–2014). Overall, our results agreed with their findings because the majority (~50%) of our sampled trees are from Orchard 1 (‘P16.013’, ‘P13.019’, and ‘Lonestar’) with continuous increments of canopy fPAR over the season. The different changing patterns of the different varieties could also be attributed to their seasonal calendars. For example, as shown in Figure 2, the full bloom date and commercial harvest date for ‘Nonpareil’ (in Orchard 2) were on February 23 and August 20 in 2019, respectively. Most other varieties had the full bloom 1–6 days later and were harvested 2–4.5 weeks later than ‘Nonpareil’.

#### VO fPAR vs. Lightbar fPAR

We compared the correlations between VO and lightbar estimated fPAR over the entire growing season in 2019 at the per-row level. Table 3 shows the overall comparison of *R*^2^, RMSE (to the 1:1 line), and the regression line slopes when we force a zero intercept. *R*^2^ and RMSE are improved from May to June/July, while they remained the same level from the June/July to August (harvest). For example, they were 0.81 and 4.06%, respectively, when we tested all varieties on May 28. In June, *R*^2^ and RMSE improved to 0.91 and 2.94%, respectively, when the date of VO data collection was within a couple of days of lightbar data collection. When we tested ‘Nonpareil’ only, we observed similar patterns but with greater *R*^2^ (0.95–0.96) and smaller RMSE (2.06–2.27%). It can be inferred that both VO and lightbar methods measured canopy light interception similarly. So, we considered a hypothetical 1:1 correlation between the VO and lightbar measurements. All slopes of the regression lines were significantly different from the 1:1 line (95% confidence level) except for July 26, ‘Nonpareil’. We then used different regression slopes for developing adjusted models for potential yield prediction using Equation 6 and summarized the results in Table 4. Comparing the regression line slopes to the 1:1 line in different days also indicated that the VO and lightbar measurements were better correlated when both data were collected within a few days. Therefore, we further calibrated and visualized the VO and lightbar estimated fPAR data in June.

**Table 3:** Comparison of *R*^2^, root mean square error (RMSE) (to the 1:1 line), and the regression line slopes (when forcing a zero intercept) between the Virtual Orchard (VO) estimated canopy fractional PAR (fPAR) and mobile lightbar estimated midday canopy fPAR (June 26) over the season in 2019 at the per-row level.

**Variety**	**May 28**			**June 26**			**July 26**			**August 7**		
	***R*^**2**^**	**RMSE (%)**	**Slope**	***R*^**2**^**	**RMSE (%)**	**Slope**	***R*^**2**^**	**RMSE (%)**	**Slope**	***R*^**2**^**	**RMSE (%)**	**Slope**
All	0.81	4.06	0.89^a^	0.91	2.94	0.91^a^	0.91	3.27	0.92^a^	0.92	2.84	0.90^a^
‘Nonpareil’	0.95	2.27	0.87^a^	0.96	2.06	0.90^a^	0.93	3.06	0.91	0.95	2.28	0.91^a^

a *Slopes are significantly different from 1:1 line using linear regression analysis (95% confidence level)*.

**Table 4:** Adjusted models for almond potential yield prediction using Virtual Orchard (VO) over the season in 2019 using two base ratios: 57.90 for all varieties and 57.70 for ‘Nonpareil’ only using a lightbar Jin et al. (2020) in June.

**Variety**	**Model**	**VO**			
		**May 28**	**June 26**	**July 26**	**August 7**
All	Slope^a^	0.89	0.91	0.92	0.90
	Adjusted ratio for new models^b^	51.53	52.69	53.27	52.11
‘Nonpareil’	Slope	0.87	0.90	0.91	0.91
	Adjusted ratio for new models	50.20	51.93	52.51	52.51

a *Slopes are retrieved from Table 3 for each date.*

b *Calculated using Equation (6)*.

**Figure 4:**
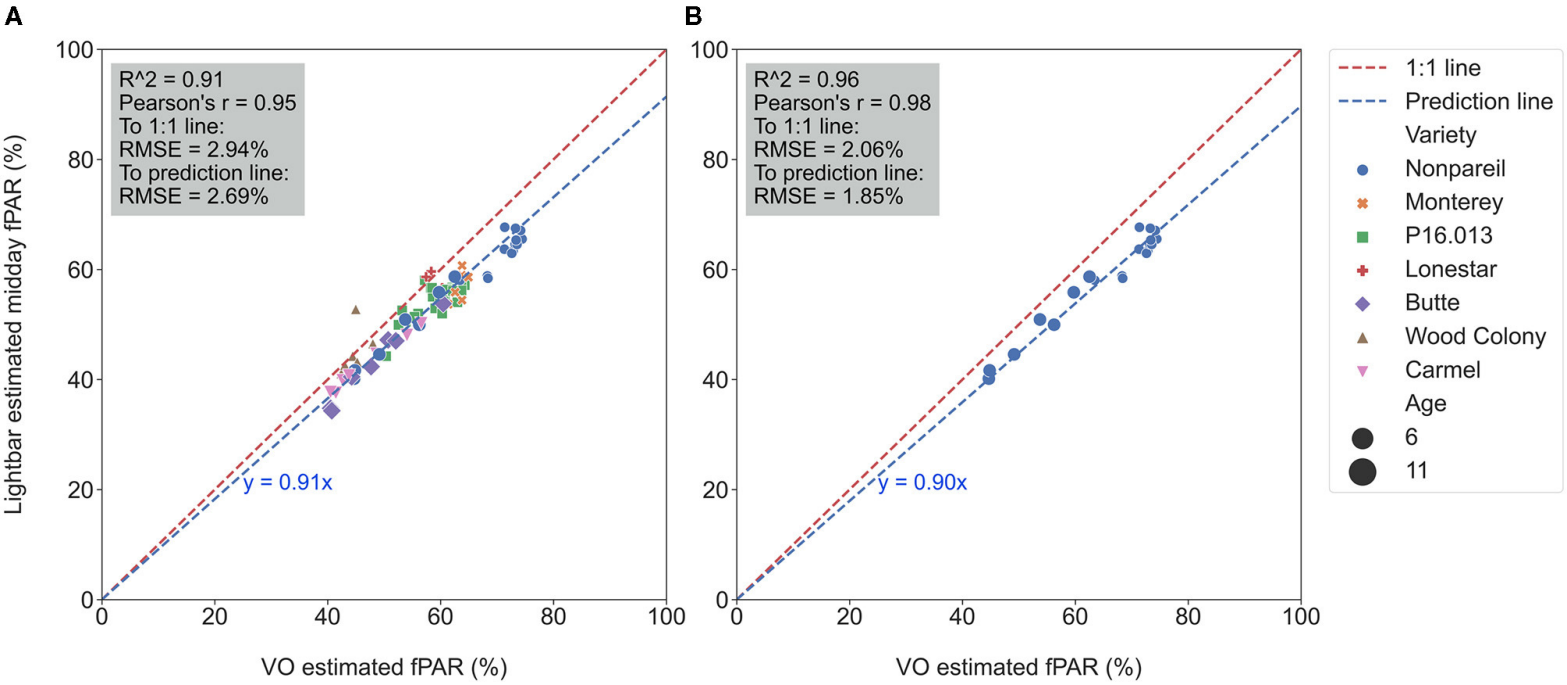
Calibration of Virtual Orchard (VO) against mobile lightbar platform (both on June 26) estimated canopy fractional PAR (fPAR) with all varieties **(A)** and ‘Nonpareil’ only **(B)** at the per-row level (when forcing a zero intercept).

Figure 4A visualizes the calibration of the VO estimated fPAR against the lightbar estimated midday fPAR over all varieties on June 26. Again, reasonably high *R*^2^ (0.91) and low RMSE (2.94%) from data points to the 1:1 line suggested that both methods estimate the actual canopy fPAR. However, one method estimated fPAR with lower accuracy or more error since some data points were slightly off the 1:1 line (in the dashed red). For instance, we observed that the regression line (in the dashed blue) was not completely overlapped with the 1:1 line with the slope of 0.91 in the clockwise direction of rotation (the difference was significant as shown in Table 3), indicating that most of the data points were below the 1:1 line. The result suggested that the lightbar tended to underestimate the canopy fPAR or the VO method tended to overestimate it (mean of absolute errors to the regression line: 1.97 ± 1.85%; to the 1:1 line: 5.09 ± 2.55%). Over different almond varieties, 6-year-old ‘Nonpareil’ trees intercepted more fPAR than others, which is reasonable because of their larger canopy size (up to 73% fPAR). Figure 4B visualized a similar calibration for ‘Nonpareil’ only with a better *R*^2^ of 0.96 and a lower RMSE of 2.06% to the 1:1 line (the difference was significant as shown in Table 3). This improved correlation might be due to the uniformity of the tree canopy from a single variety (mean of absolute errors to the regression line: 1.58 ± 1.00%; to the 1:1 line: 6.59 ± 2.47%). We scanned all three orchards using the aerial photogrammetry method within a day for each data collection from May to August. However, the lightbar method needed multiple days to complete the same tasks. Therefore, the VO method we offered in this study is practically easier and faster to conduct than a mobile lightbar platform to estimate canopy fPAR in the orchard environment.

**Figure 5:**
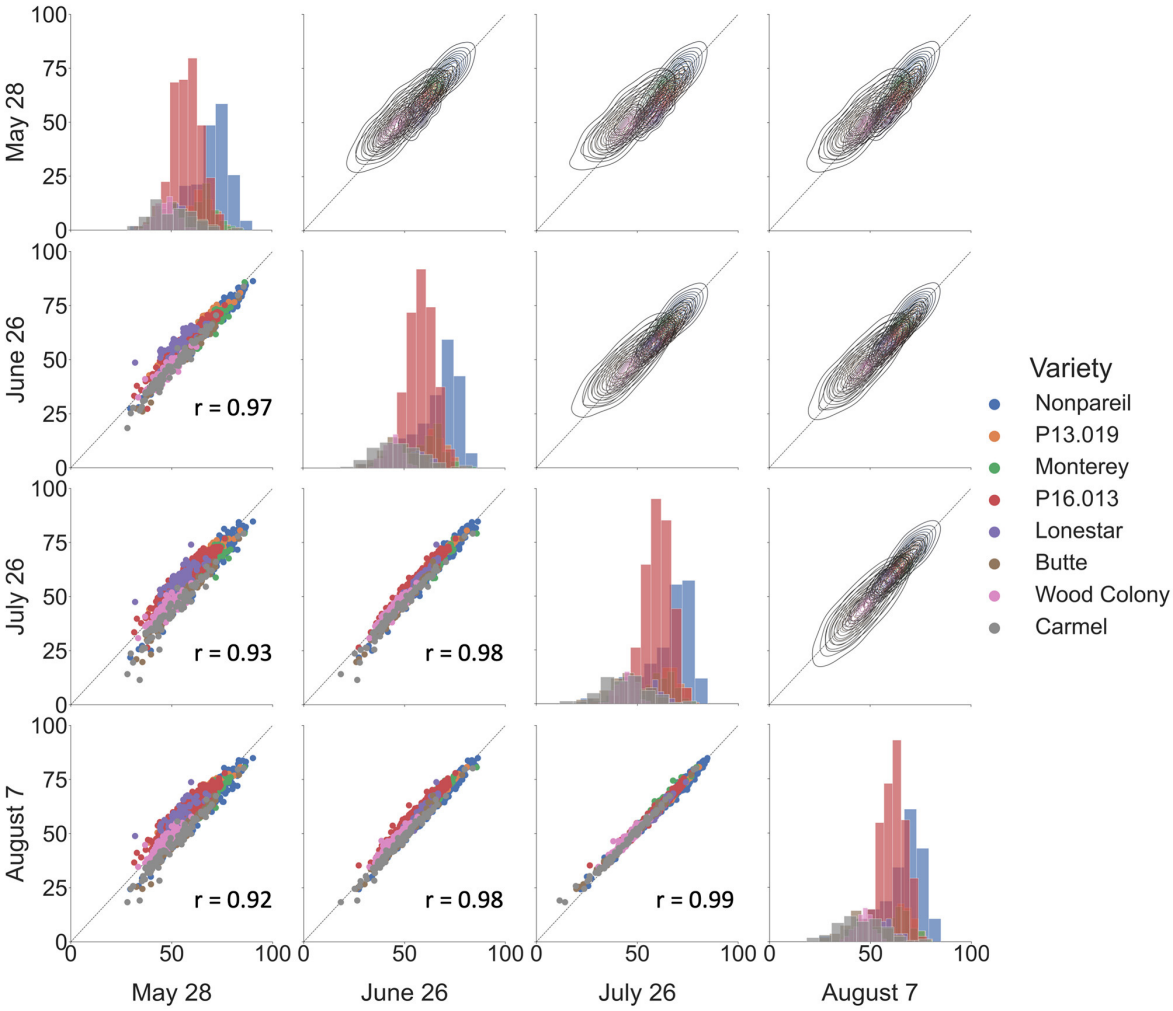
Correlations of Virtual Orchard (VO) estimated canopy fractional PAR (fPAR) over the growing season in 2019. Scatter plots are shown in the lower-left half, kernel–density–estimate plots are shown in the upper right half, and histograms are shown on the diagonal. The black dotted line indicates the 1:1 line. r refers to Pearson's correlation coefficient.

Figure 5 shows the overall correlations of VO estimated fPAR over the growing season in 2019. We observed a good correlation among the data collected at different dates, with Pearson's r ranged between 0.92 and 0.99. Based on the scatter plots in the lower-left half, we could find better correlations when the two dates were closer. The highest Pearson's r of 0.99 was from the dates between July 26 and August 7. One possible reason could be the proximity of data collection dates (only 10-d difference). Besides, the histograms on the diagonal illustrate that all sampled trees were normally distributed, and the majority of the sampled trees were ‘Nonpareil’ and ‘P16.013’. Lastly, the kernel-density-estimate plots in the upper right half show that the variations of VO estimated fPAR decreased from May to August with more data points aligned on the 1:1 line. Figure 5 illustrates the results of 12 different flights, image processing, and feature extractions conducted by the VO pipeline. High correlations in Figure 5 show the robustness and replicability of the VO methodology that consistently and accurately estimated fPAR in all 12 datasets. While we observed some canopy development over the season that leads to minor changes in the canopy profile, no major observational error (variation in measurement) was noticed for the same tree profile features measured during the season. To explore which method is more reliable, we further compared the VO and lightbar methods in estimating the actual almond yield from different spatial precision levels: per-row, per-block, and per-tree (VO only) level.

### Actual Almond Yield Estimation

#### Per-Row Analysis

**Figure 6:**
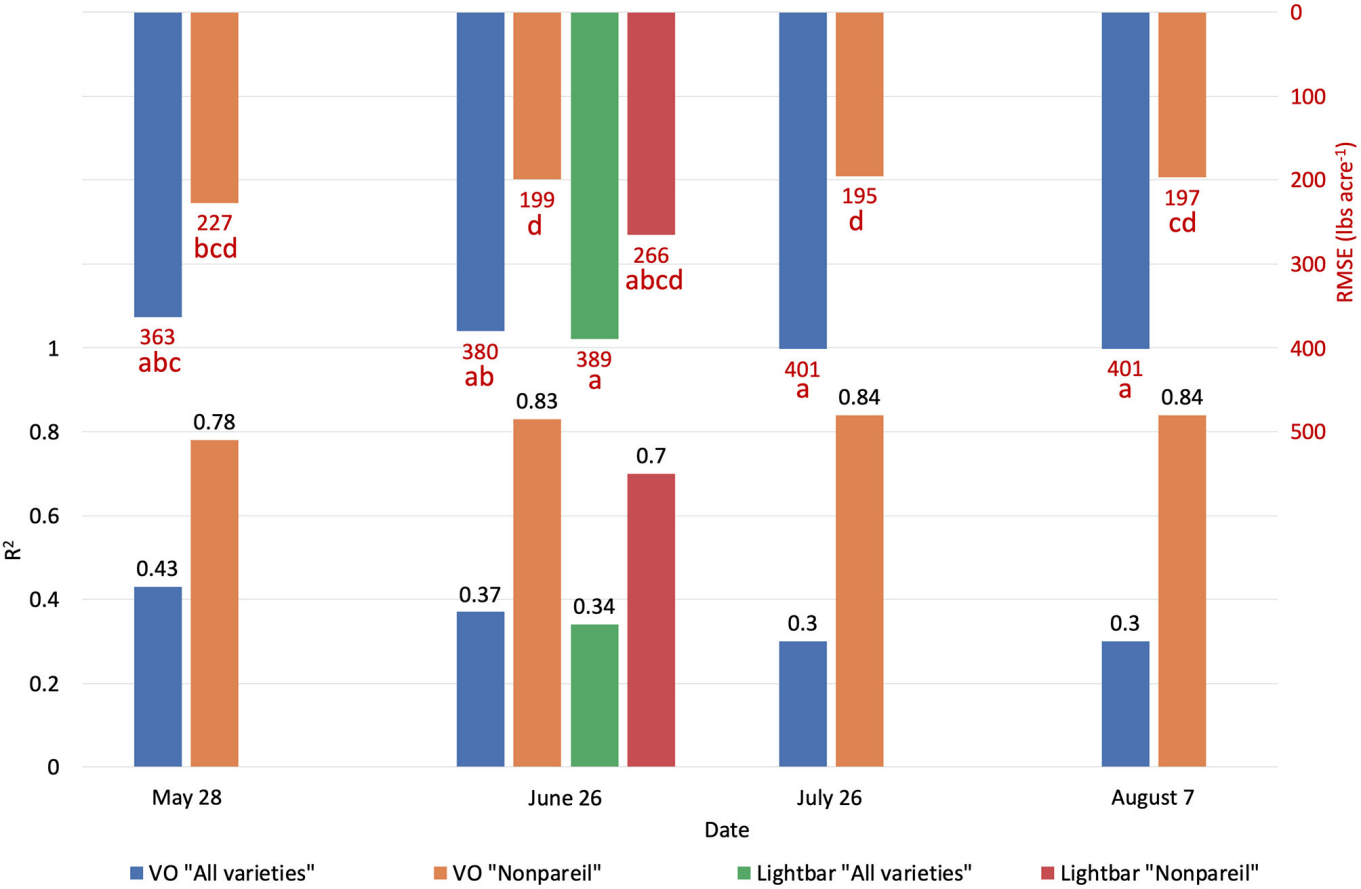
Comparison of *R*^2^ and root mean square error (RMSE) (to regression line) between Virtual Orchard (VO) and the mobile lightbar platform estimated canopy Fractional PAR (fPAR) and actual almond kernel yield for all varieties and ‘Nonpareil’ only over the season in 2019 at the per-row level. Different letters refer to a statistically significant difference testing the means of absolute errors in predicting actual almond yield with different regression models using analysis of variance (ANOVA) multi-comparison (*p* < 0.05).

**Figure 7:**
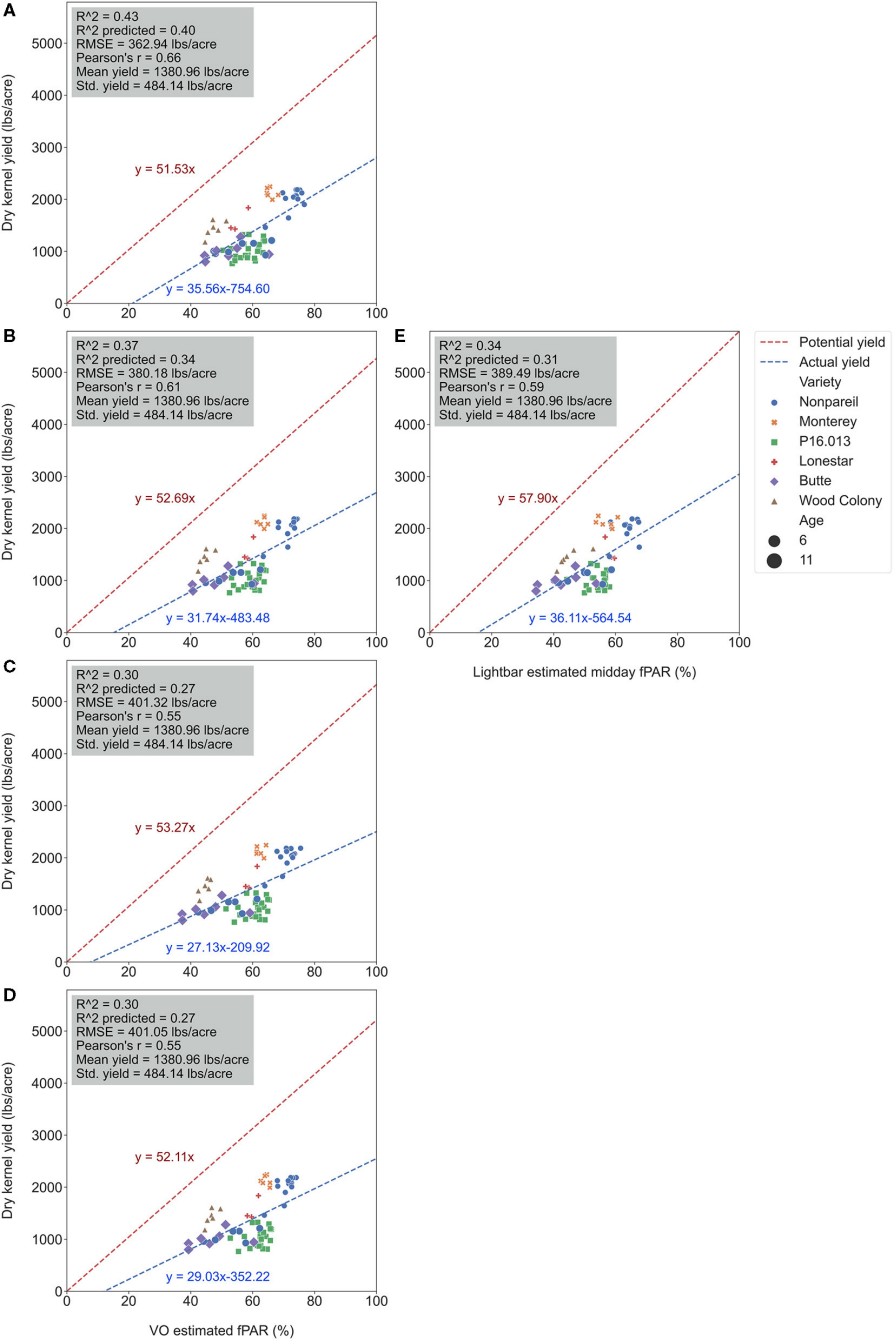
Comparison of accuracies of actual almond yield explained by Virtual Orchard (VO) estimated canopy Fractional PAR (fPAR) over the season: May 28 **(A)**, June 26 **(B)**, July 26 **(C)**, August 7 **(D)**, and the mobile lightbar platform (June 26) **(E)** at the per-row level in 2019. The potential almond yield (57.9 lb acre^−1^) was based on the results reported by Jin et al. (2020) using a lightbar.

To test which method could better estimate actual almond yield, we compared the scatter plots of actual yield vs. canopy fPAR estimated by the mobile lightbar and VO methods on a per-row basis. Figure 6 summarizes the overall results, and Figure 7 shows more details in Supplementary Material. The results indicated that canopy fPAR estimated by VO is a more accurate indicator of almond actual yield than lightbar. Both *R*^2^ and predictive *R*^2^ are higher with the VO method (0.37 and 0.34 for VO; 0.34 and 0.31 for lightbar) on June 26. Both regression lines in Figures 7B,E had similar slopes (32 for VO; 36 for lightbar) and intercept on the y-axis. We may accept either method since both RMSEs were lower than the standard deviation of the actual yield (484.14 lb acre^−1^) with all varieties. We did not find any statistical difference between the means of absolute errors in predicting actual almond yield by the VO and mobile lightbar models. So, the VO method is statistically as good as the lightbar measurement but with potentially better performance as the VO models presented insignificantly smaller errors. One possible reason might be the higher spatial resolution of the VO method (0.01–0.02 m) compared with the mobile lightbar (~0.4 m). In general, the *R*^2^ of 0.34 estimated by the lightbar in our study agreed with the findings from Zarate-Valdez et al. (2015), who reported that mobile lightbar estimated midday fPAR was an indicator of almond kernel yield with *R*^2^ in the range of 0.16–0.36. A better correlation between VO estimated fPAR suggested that light interception, or fPAR, is not only a good indicator of potential (maximum) yield but also a possible good estimator of actual yield for almond (particularly the ‘Nonpareil’ variety).

We conducted separate analytics for ‘Nonpareil’ since it is the leading and most profitable almond variety in California. Results indicated better fits for both models if we only analyze the ‘Nonpareil’ variety. For example, we achieved a better *R*^2^ of 0.83 and predictive *R*^2^ of 0.79 for the ‘Nonpareil’ yield prediction than the model for all varieties (0.37 and 0.34, respectively). One reason might be that the sampled trees are more uniform when we tested with only one variety. This better performance on the ‘Nonpareil’ variety is also valid for using lightbar estimated midday fPAR. However, the achieved *R*^2^ (0.70) and predictive *R*^2^ (0.65) are notably lower than those from VO. Pearson's r followed a similar pattern. RMSEs for both methods with ‘Nonpareil’ only were lower than the standard deviation (497.64 lb acre^−1^) of actual almond yield at the per-row level. However, the RMSE of VO estimated fPAR (199.26 lb acre^−1^) was notably smaller than the mobile lightbar (265.73 lb acre^−1^) in June. These results reiterated the possibility that fPAR alone could be an accurate indicator of actual almond yield if it is estimated accurately. We may accept both VO and lightbar results in explaining ‘Nonpareil’ yield, but apparently, the result from VO seemed more accurate and reliable. Overall, we suggest that the VO method offers a more accessible and accurate (with smaller errors) alternative for estimating canopy fPAR than the mobile lightbar platform since the VO model showed a better correlation to actual yield. Therefore, we can use this VO technology as a reliable indicator for predicting actual almond yield, particularly with the ‘Nonpareil’ almond variety.

In addition to comparing VO estimated fPAR with lightbar data, we compared the yield prediction models based on data at a different time of the season. Interestingly, a descending trend of yield prediction accuracy is present from May to August when we tested all varieties (Figure 6). In other words, we found that we achieved a better actual yield prediction in May comparing to June, July, or August using VO estimated fPAR model for all varieties. The overall trend was in a reverse direction when we only tested ‘Nonpareil’; the prediction accuracy on ‘Nonpareil’ improved as we got closer to the harvest time in August. This discrepancy of accuracy changes between all varieties and ‘Nonpareil’ suggests that a single comprehensive model cannot accurately predict yield for different almond varieties, and the yield prediction model should be calibrated for each variety. It might be more reasonable to develop models separately for each variety, such as ‘Nonpareil’, to explain the variations of actual almond yield.

The yield prediction accuracy with VO for ‘Nonpareil’ improved from May to June but did not change much after June 26. The source–sink interaction theory during plant growth may better explain it (Allen et al., 2005). Starting from the bloom in almonds (around February in a year), the reproductive growth (with reproductive organs: flower and fruit) is becoming more competitive with vegetative growth (with vegetative organs: branches and leaves) for absorbing the nutrients. Therefore, midseason (June/July) measurement might be more accurate for predicting actual almond yield when both growths are dominant. This deduction was verified because the midday fPAR was mostly measured with a lightbar system in midseason (June and July) for almond orchards (Lampinen et al., 2012; Rubke, 2015). Lastly, the similarity of actual yield prediction results on June 26 between the lightbar (*R*^2^ = 0.34 for all varieties; *R*^2^ = 0.70 for ‘Nonpareil’ only) and VO method (*R*^2^ = 0.37 for all varieties; *R*^2^ = 0.83) also verified the strong correlation between these two methods of fPAR measurement as discussed earlier.

**Figure 8:**
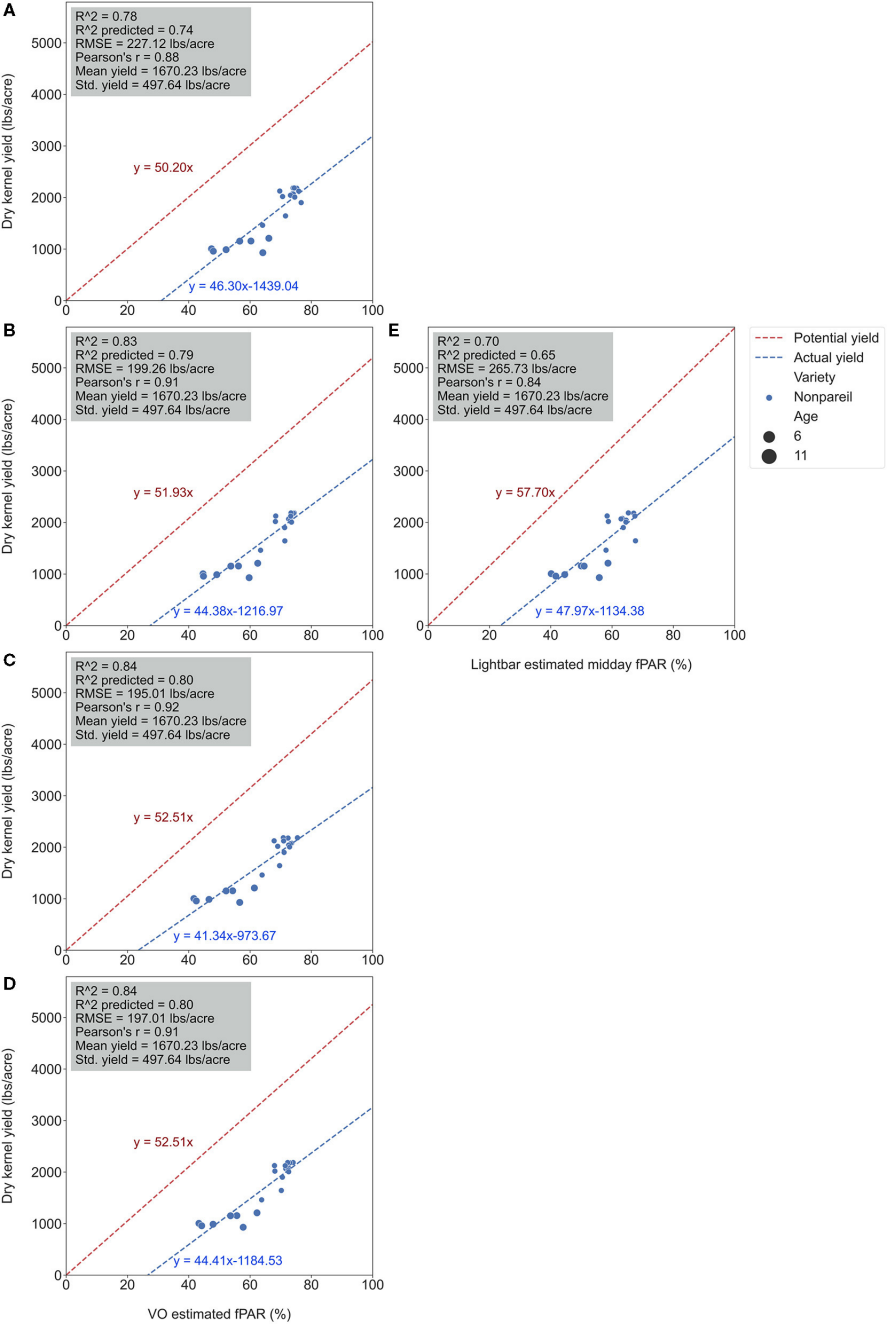
Comparison of accuracies of actual ‘Nonpareil’ almond yield explained by Virtual Orchard (VO) estimated canopy Fractional PAR (fPAR) over the season: May 28 **(A)**, June 26 **(B)**, July 26 **(C)**, August 7 **(D)**, and the mobile lightbar platform (June 26) **(E)** at the per-row level in 2019. The potential almond yield (57.7 lb acre^−1^) was based on the results reported by Jin et al. (2020) using a lightbar.

It was also interesting to notice that the regression lines (dashed blue line in Figure 8) had a smaller angle to the 1:1 line for ‘Nonpareil’ than all varieties combined in Figure 7. First of all, all data points (at the per-row level in Figure 7) were below the 1:1 line, indicating that none of the samples reached the potential yield in this study. Specifically, with the increase of estimated fPAR on the x-axis (0–100%), the slope of the regression lines moved farther (35.6 in Figure 7A to 29.0 in Figure 7D) from the potential yield line over the season for all varieties, suggesting that the actual yield prediction became less accurate. Comparatively, the regression lines directions were closer to the 1:1 line for ‘Nonpareil’ only (Figures 8A–D). Although the actual yield of ‘Nonpareil’ was also far less than the potential yield, the changing patterns were more comparable. We believe that the proposed model could be used as a good yield forecasting tool for ‘Nonpareil’ trees with an acceptable accuracy of *R*^2^ = 0.84 and RMSE = 195 lb acre^−1^ at the per-row precision level, where the standard deviation of the yield (498 lb acre^−1^) was about 2.5 times greater than RMSE. Besides, we analyzed the VO estimated fPAR with higher spatial precision (at the per-tree level) in the next section, but no corresponding lightbar data at this level of precision was available for comparison.

#### Per-Tree Analysis

**Figure 9:**
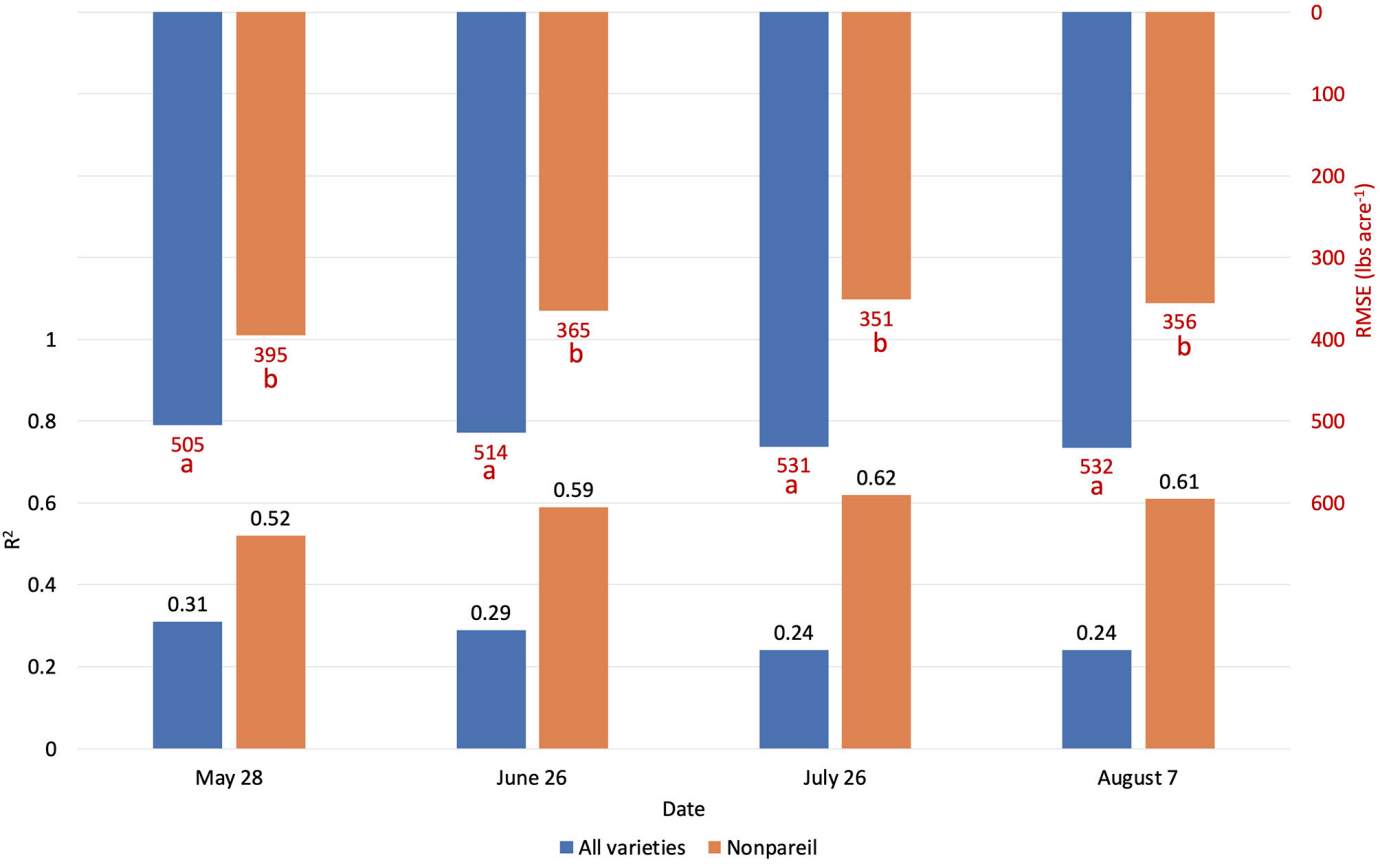
Comparison of *R*^2^ and root mean square error (RMSE) (to regression line) between fPAR Virtual Orchard (fractional PAR estimated by VO) and actual almond kernel yield for all varieties and ‘Nonpareil’ only over the season in 2019 at the per-tree level. Different letters refer to a statistically significant difference testing the means of absolute errors in predicting actual almond yield with different regression models using analysis of variance (ANOVA) multi-comparison (*p* < 0.05).

**Figure 10:**
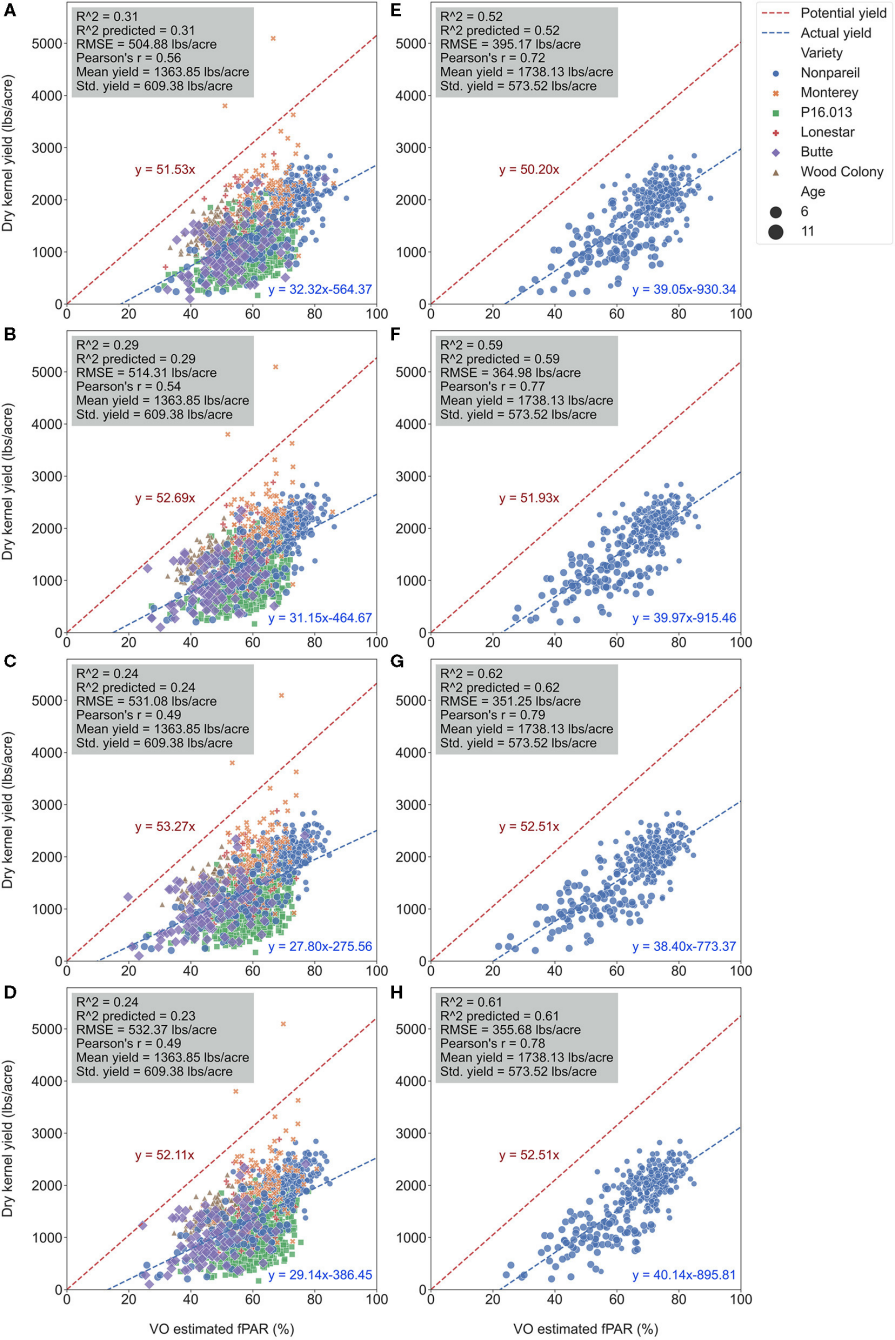
Comparison of accuracies of actual almond yield explained by Virtual Orchard (VO) estimated canopy fractional PAR (fPAR) over the season with all varieties: May 28 **(A)**, June 26 **(B)**, July 26 **(C)**, and August 7 **(D)**; and ‘Nonpareil’ only: May 28 **(E)**, June 26 **(F)**, July 26 **(G)**, and August 7 **(H)** at the per-tree level in 2019. The potential almond yield (57.9 lb acre^−1^ for all varieties; 57.7 lb acre^−1^ for ‘Nonpareil’ only) was based on the results reported by Jin et al. (2020) using a lightbar.

Figure 9 visualizes the regressions between per-tree fPAR (estimated by VO) and almond kernel yield over the season in 2019. Figure 10 provides in-depth details. As discussed for the per-row level results, the accuracy (*R*^2^) decreased for all-varieties-model from May (May 28; with *R*^2^ of 0.31 and RMSE of 504.88 lb acre^−1^) to August (August 7; with *R*^2^ of 0.24 and RMSE of 532.37 lb acre^−1^). The result at the per-tree level had a relatively poorer *R*^2^ (0.29), and RMSE (514.31 lb acre^−1^) with a greater standard deviation of the yield (609.38 lb acre^−1^) compared with the per-row analysis (*R*^2^ = 0.37 and RMSE = 38.18 lb acre^−1^ for VO; *R*^2^ = 0.34 and RMSE = 389.49 lb acre^−1^ for lightbar; the standard deviation was 484.14 lb acre^−1^) in June. This result is reasonable because we expected a greater variation in the dataset for the high-spatial-precision level of per-tree data. For instance, we observed a huge range of yield from 10.81 to 5094.49 lb acre^−1^ at a per-tree precision level. Given this large variation in per-tree yield data, VO estimated fPAR still offered a reasonable accuracy with higher spatial precision than lightbar data, which is mainly available at the per-row level.

When we only considered the ‘Nonpareil’ variety, we observed an increasing trend in accuracy (*R*^2^) similar to the per-row models (Figure 6). On July 26, the *R*^2^ was up to 0.62, and RMSE was 351.25 lb acre^−1^ for the ‘Nonpareil’ per-tree yield prediction. As expected, these results were not as good as the per-row model (*R*^2^ = 0.84 and RMSE = 195.01 lb acre^−1^). The RMSE from per-tree data were also much greater than that from per-row data due to the more diversity in the yield data. The differences were only significant between the two scenarios (i.e., all varieties and ‘Nonpareil’) when we tested the means of absolute errors using ANOVA (*p* < 0.05). In general, we conclude that the VO method provides an acceptable yield prediction accuracy (*R*^2^) at the per-tree level; a level of spatial precision in yield forecasting that has not been offered by the mobile lightbar platform or any other yield prediction methodologies.

It was important to note that more scattered points were getting closer to the potential yield line (Jin et al., 2020) due to the more variation in per-tree yield data. Two ‘Monterey’ trees (in yellow “x” symbol) yielded (5094.49 and 3801.04 lb acre^−1^) more than their potential yields (i.e., the two symbols were above the potential line in Figures 10A–D). Jin et al. (2020) found that the variation of the almond yield gap from actual yield to the potential yield (pass or not reach) was mainly driven by tree age and other factors if the orchards were located in different geographical areas. Since our three orchards were located close to each other, and all trees were mature, this gap was probably triggered by different orchard management and practices.

#### Comparison of Spatial Precision Levels

**Figure 11:**
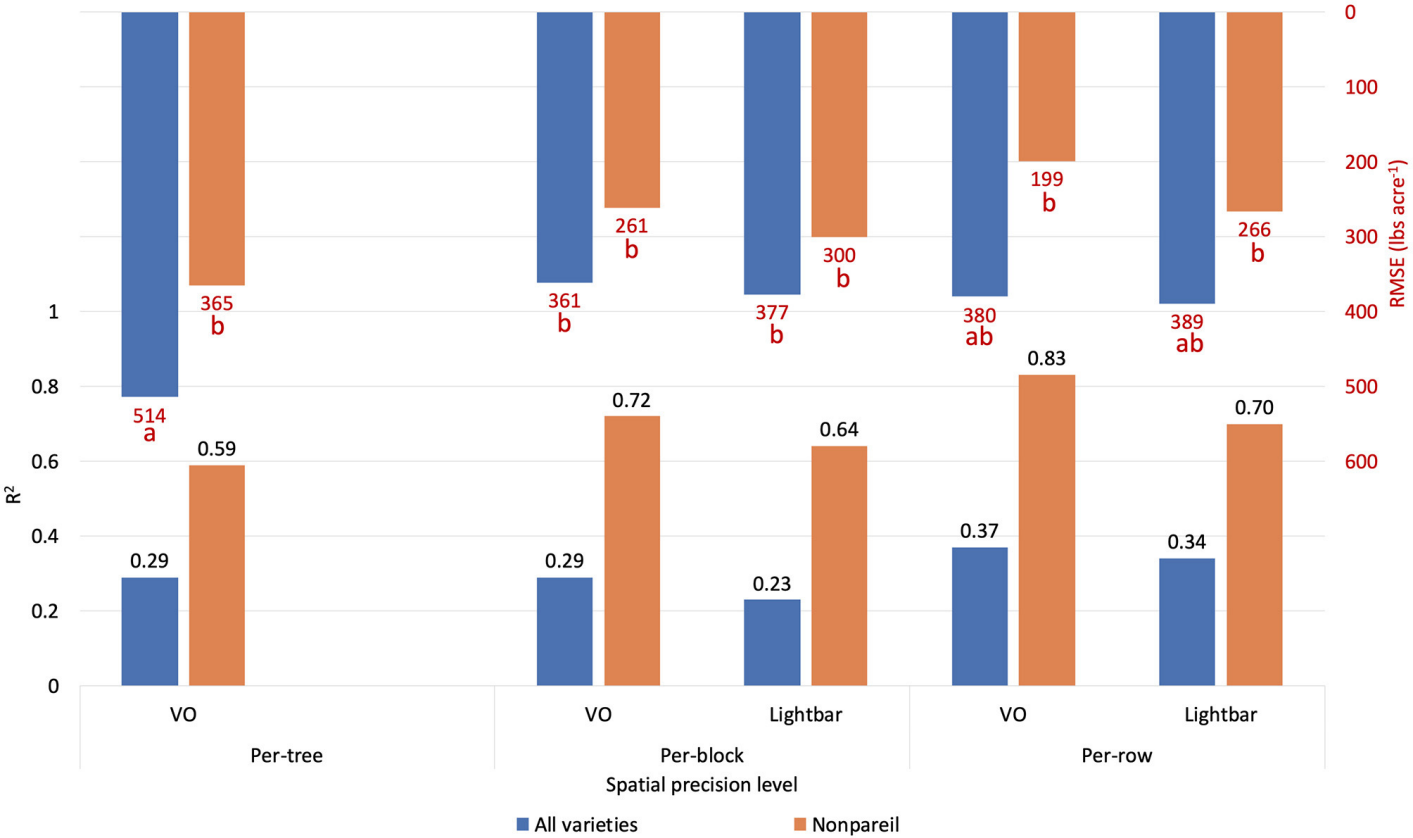
Comparison of *R*^2^ and root mean square error (RMSE) (to regression line) between fPAR Virtual Orchard (fractional PAR estimated by VO) and actual almond kernel yield for all varieties and ‘Nonpareil’ only at the per-tree, per-block, and per-row spatial precision levels. Both VO and lightbar data were collected on June 26. Different letters refer to a statistically significant difference testing the means of absolute errors in predicting actual almond yield with different regression models using analysis of variance (ANOVA) multi-comparison (*p* < 0.05).

Orchards 1 and 3 contain several blocks in each row, consisting of three to eight trees based on the orchard configuration. Therefore, we also analyzed the data based on the per-block level, where the lightbar data were available to compare. Figure 11 compares the accuracies (*R*^2^) of the different measuring levels [per-tree (VO only), per-block, and per-row] in explaining almond actual kernel yield on June 26. In general, as the precision level decreased from per-block to per-row, the accuracy (*R*^2^) increased for both methods. For example, *R*^2^ increased from 0.29 to 0.37 using VO estimated fPAR in June. We expected this trend since the dataset contained more errors or extreme values when the precision level was higher. Subtle changes, such as a tree being shaded from neighboring trees, can cause biased data collection. We observed that even with the highest level of precision (at the per-tree level), the VO method achieved better accuracies (*R*^2^) than that of lightbar at a lower level of precision (at per-block level) for all varieties (*R*^2^ = 0.29 for VO at tree level; *R*^2^ = 0.23 for lightbar at block level). In other words, VO estimated fPAR could better explain the actual kernel yield with both higher accuracy and spatial precision than lightbar. Regarding ‘Nonpareil’, the per-tree VO method (*R*^2^ = 0.59) performed only slightly worse than the lightbar at the per-block level (*R*^2^ = 0.64). Overall, we can conclude that the correlation (*R*^2^) between the two measures (i.e., VO and lightbar estimated fPAR) can be up to 0.96, in which the VO method even better explained the almond actual kernel yield with up to 0.83 of *R*^2^ on the leading variety of ‘Nonpareil’ in June. The estimation accuracy was further improved for ‘Nonpareil’ toward the end of the season using the VO data.

In future work, we will consider adopting the DSM model generated by the LiDAR sensor instead of RGB photogrammetry, where LiDAR has much higher pixel resolutions. It is expected that fractional absorbed PAR (fAPAR) is highly correlated to fPAR due to fAPAR is strongly correlated to transmittance. In the PAR domain, the fAPAR is very close to the fPAR, corresponding to the canopy transmittance in the sun direction (Todd et al., 2003). Although only fPAR was considered in this work, we already tested the relationship between accumulated PAR (in the unit of mole) and fPAR using some preliminary datasets, where sun angle and sunlight directions were precisely modeled from sunrise to sunset at the specific locations of almond orchards. Per accumulating the total PAR over the entire growing season (four months from April 1 to July 31), we achieved a very high correlation (*R*^2^ = 0.97–0.99) between accumulated PAR and fPAR. Such information and results are out of the scope of this work and, therefore, will be presented in our future publications. We will also take the nitrogen and irrigation scheduling effects on the plants into account when we evaluate the correlation between estimated fPAR and almond yield. Besides, we will consider employing spectral reflectance (Moghimi et al., 2020) to determine tree nitrogen status and to better explain the yield variations in trees of similar size.

## Conclusions

In this study, a complete processing pipeline called VO was developed in a Python environment to accurately extract canopy profile features (e.g., canopy cover and canopy volume index) from user-input orchards and user-defined parameters. Midday canopy fPAR estimated by a mobile lightbar platform from each row was compared against the canopy fPAR estimated by VO in midseason (June) for eight different almond varieties, including California's leading variety of ‘Nonpareil’. The temporal analysis was also conducted for VO estimated fPAR throughout the entire growing season in 2019. Finally, regression models were established for predicting actual almond yield based on VO estimated canopy fPAR from different spatial precision levels. Specific conclusions from this study are presented as follows:

- We achieved a strong correlation (*R*^2^) of 0.91 and a low RMSE of ~3% between the VO and lightbar estimated fPAR in midseason (June) for all varieties; the results were further improved when we tested ‘Nonpareil’ only with an *R*^2^ of 0.96 and RMSE of 2%. In addition to June, *R*^2^ was ranged 0.81–0.92 for all varieties and 0.93–0.95 for ‘Nonpareil’ in May and August (harvest season) between the VO and lightbar;
- With the VO method, we achieved a better correlation (*R*^2^ of 0.43 and RMSE of 363 lb acre^−1^) in May between actual almond yield and fPAR for the all-varieties model at the row level; those numbers were 0.34 and 389 lb acre^−1^ when mobile lightbar was used in the midseason (June). When we tested ‘Nonpareil’, *R*^2^ and RMSE reached 0.84 and 195 lb acre^−1^ for VO method, and 0.70 and 266 for lightbar both in the midseason (June–July);
- We compared the two measures at different spatial precision levels: per-tree (VO only), per-block, and per-row; results indicated that the lower the precision level, the better the accuracy for both methods, and vice versa; however, with the same precision level, the VO method performed notably better than mobile lightbar (up to 0.13 higher *R*^2^).

With the results obtained from this study, we can conclude that the VO method offers a practically more accessible and more accurate, and precise alternative in estimating canopy fPAR to replace the mobile lightbar platform. Tree-to-tree variations are ready to be visualized, featured, and evaluated using our proposed approach to facilitate better decision-making for almond growers. This study showed further evidence of a fundamental link between canopy light interception (or fPAR; that can be estimated by aerial imagery and VO method) and almond yield. The findings from this work provide a solid foundation for further investigation of canopy 3D models for yield forecasting in nut crops.

## Data Availability Statement

The data analyzed in this study is available on request. Requests to access these datasets should be directed to Alireza Pourreza, apourreza@ucdavis.edu.

## Author Contributions

XZ performed data curation, investigation, formal analysis, validation, visualization, and writing of the original draft. AP performed conceptualization, supervision, funding acquisition, project administration, writing review, and editing. KC performed methodology, software, and data curation. GZ-R performed methodology, software, and data curation. BL handled resources and performed writing—review and editing. KS handled resources and performed writing—review and editing. All authors contributed to the article and approved the submitted version.

## Conflict of Interest

The authors declare that the research was conducted in the absence of any commercial or financial relationships that could be construed as a potential conflict of interest.

## Publisher's Note

All claims expressed in this article are solely those of the authors and do not necessarily represent those of their affiliated organizations, or those of the publisher, the editors and the reviewers. Any product that may be evaluated in this article, or claim that may be made by its manufacturer, is not guaranteed or endorsed by the publisher.

## Footnotes

^1^ https://rpubs.com/RatherBit/102428
