# Supplementary material for: Estimation of Fractional Photosynthetically Active Radiation From a Canopy 3D Model; Case Study: Almond Yield Prediction
Source: Front Plant Sci. 2021 Aug 26;12:715361. doi: 10.3389/fpls.2021.715361 (PMC8427806; doi:10.3389/fpls.2021.715361)
Supplement: Supplementary file 1 [file Data_Sheet_1.pdf]

Supplementary Materials

for

Estimation of Fractional Photosynthetically Active Radiation from a Canopy 3D Model; Case Study: Almond Yield Prediction

Xin Zhang[1], Alireza Pourreza[1*], Kyle H. Cheung[1], German Zuniga-Ramirez[1,2], Bruce Lampinen[3], Kenneth A. Shackel[3]

[1]Department of Biological and Agricultural Engineering, University of California, Davis, Davis, CA, USA

[2]Kearney Agricultural Research and Extension Center, University of California, Agriculture and Natural Resources, Parlier, CA, USA

[3]Department of Plant Sciences, University of California, Davis, Davis, CA, USA

*** Correspondence:**
Alireza Pourreza
apourreza@ucdavis.edu

Supplementary Figures

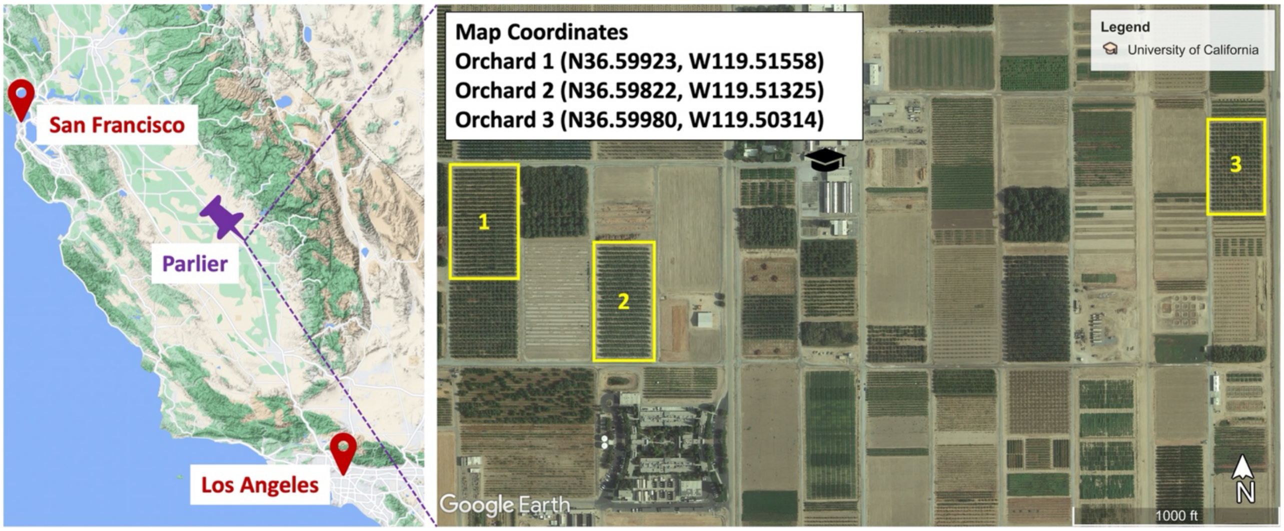

Supplementary Figure 1. Geographic locations and coordinates of the three almond orchards at the University of California Kearney Agricultural Research and Extension Center (KAREC) at Parlier, California (on Google Earth and Google Maps). The mosaics of each orchard can be found in Supplementary Figure 2.

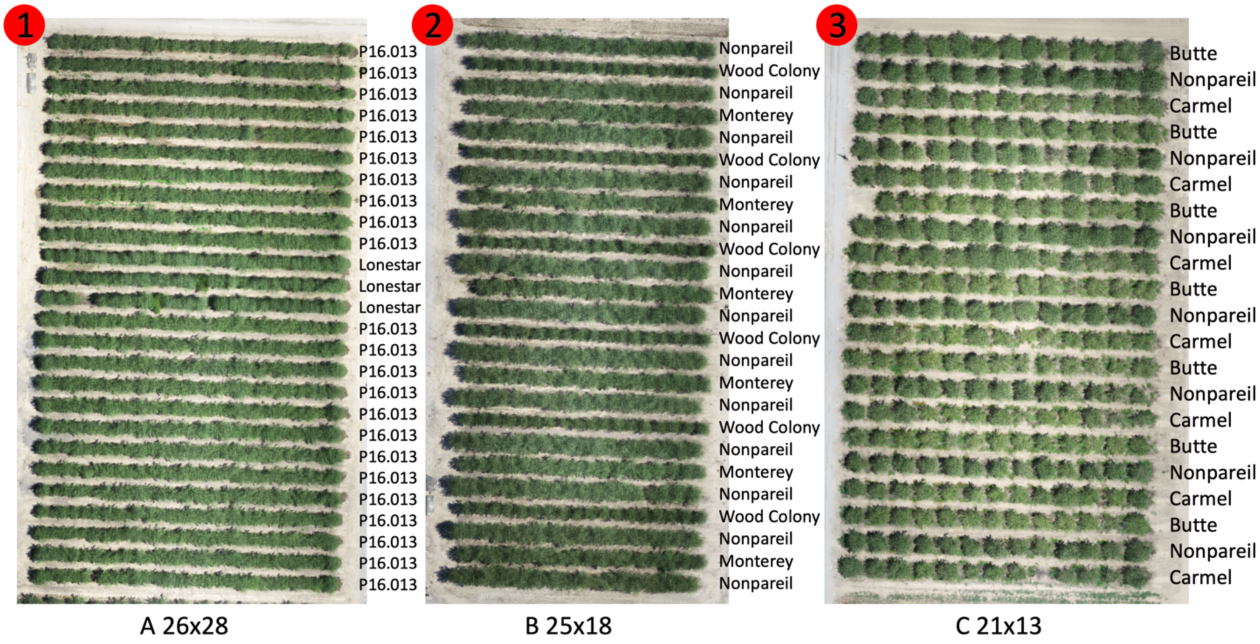

Supplementary Figure 2. Overall layouts (number of rows × number of trees) of the three orchards (orchard 1 **(A)**, 2 **(B)**, and 3 **(C)**) used in this study at the University of California Kearney Agricultural Research and Extension Center (KAREC) at Parlier, California. The almond variety names for each row are shown on the right side of the orchard's mosaic.

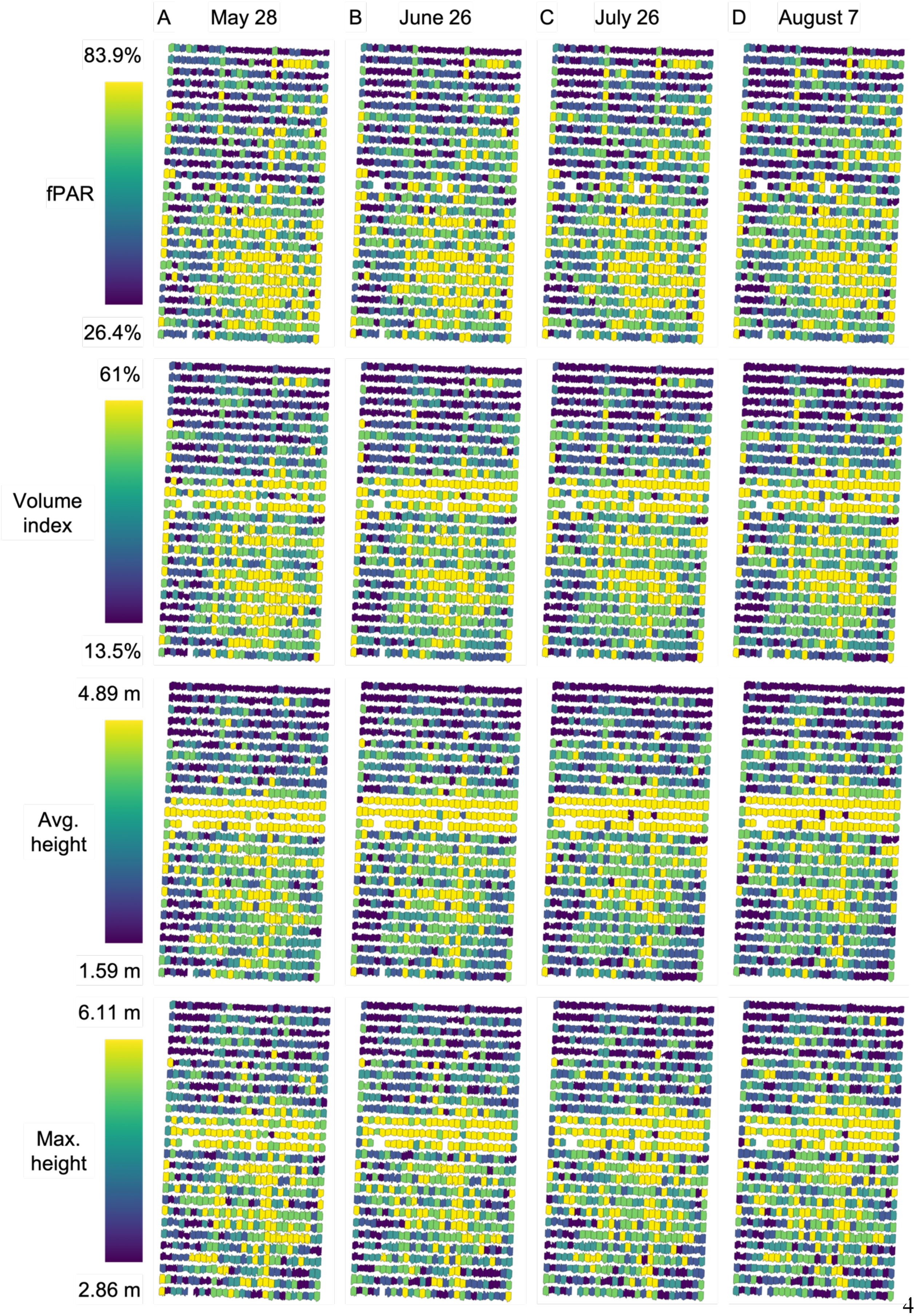
A
May 28
B
June 26
C
July 26
D
August 7
83.9%
fPAR
26.4%
61%
Volume index
13.5%
4.89 m
Avg. height
1.59 m
6.11 m
Max. height
2.86 m

Supplementary Figure 3. The visualization of all four extracted canopy profile features derived from digital surface models (DSMs), including fractional photosynthetically active radiation (fPAR), canopy volume index, average canopy height, and maximum canopy height, at the per-tree level for experimental almond orchard 1 (May 28 (**A**), June 26 (**B**), July 26 (**C**), and August 7 (**D**) in 2019).

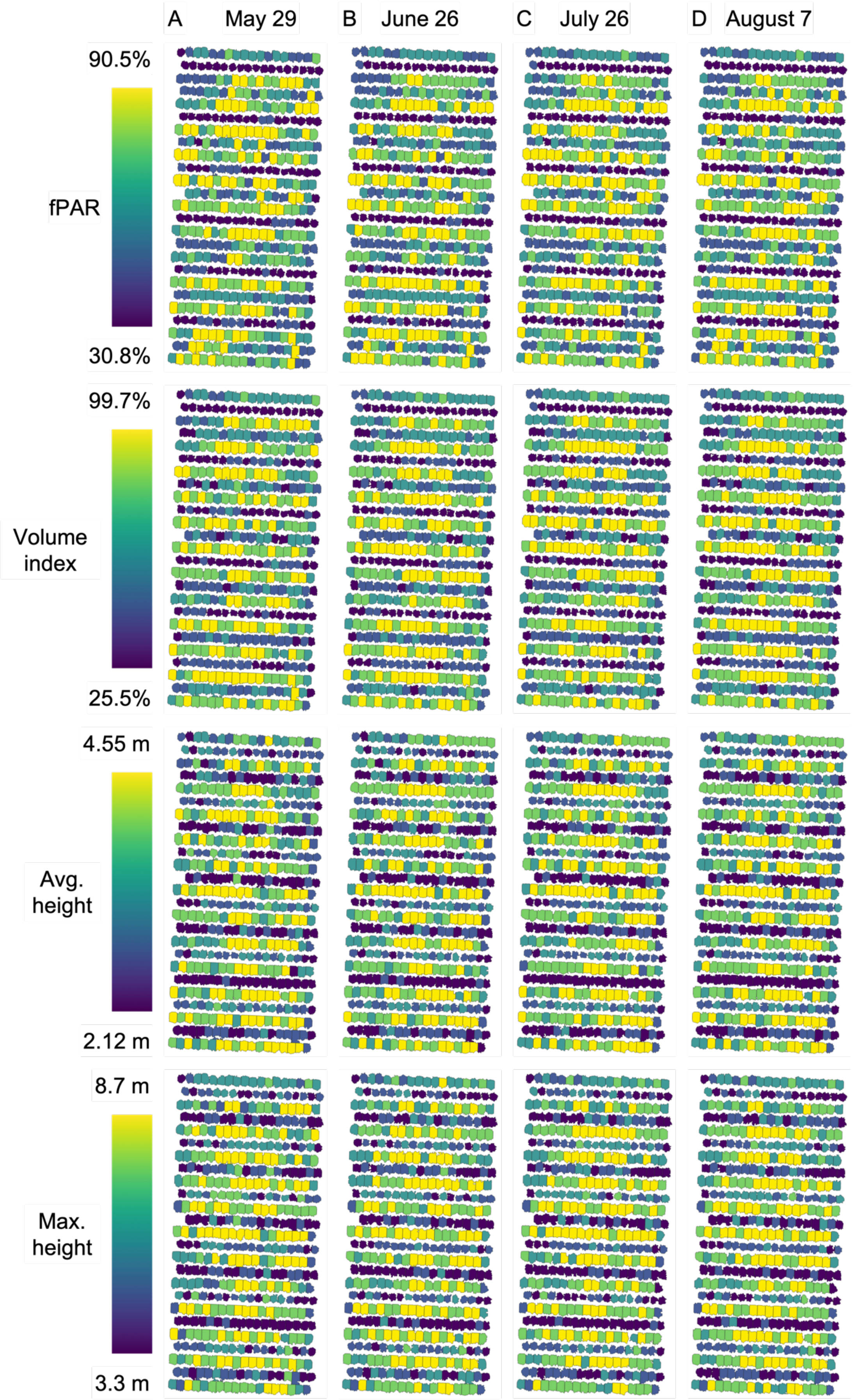
A
May 29
B
June 26
C
July 26
D
August 7
90.5%
fPAR
30.8%
99.7%
Volume index
25.5%
4.55 m
Avg. height
2.12 m
8.7 m
Max. height
3.3 m

Supplementary Figure 4. The visualization of all four extracted canopy profile features derived from digital surface models (DSMs), including fractional photosynthetically active radiation (fPAR), canopy volume index, average canopy height, and maximum canopy height, at the per-tree level for experimental almond orchard 2 (May 29 (**A**), June 26 (**B**), July 26 (**C**), and August 7 (**D**) in 2019).

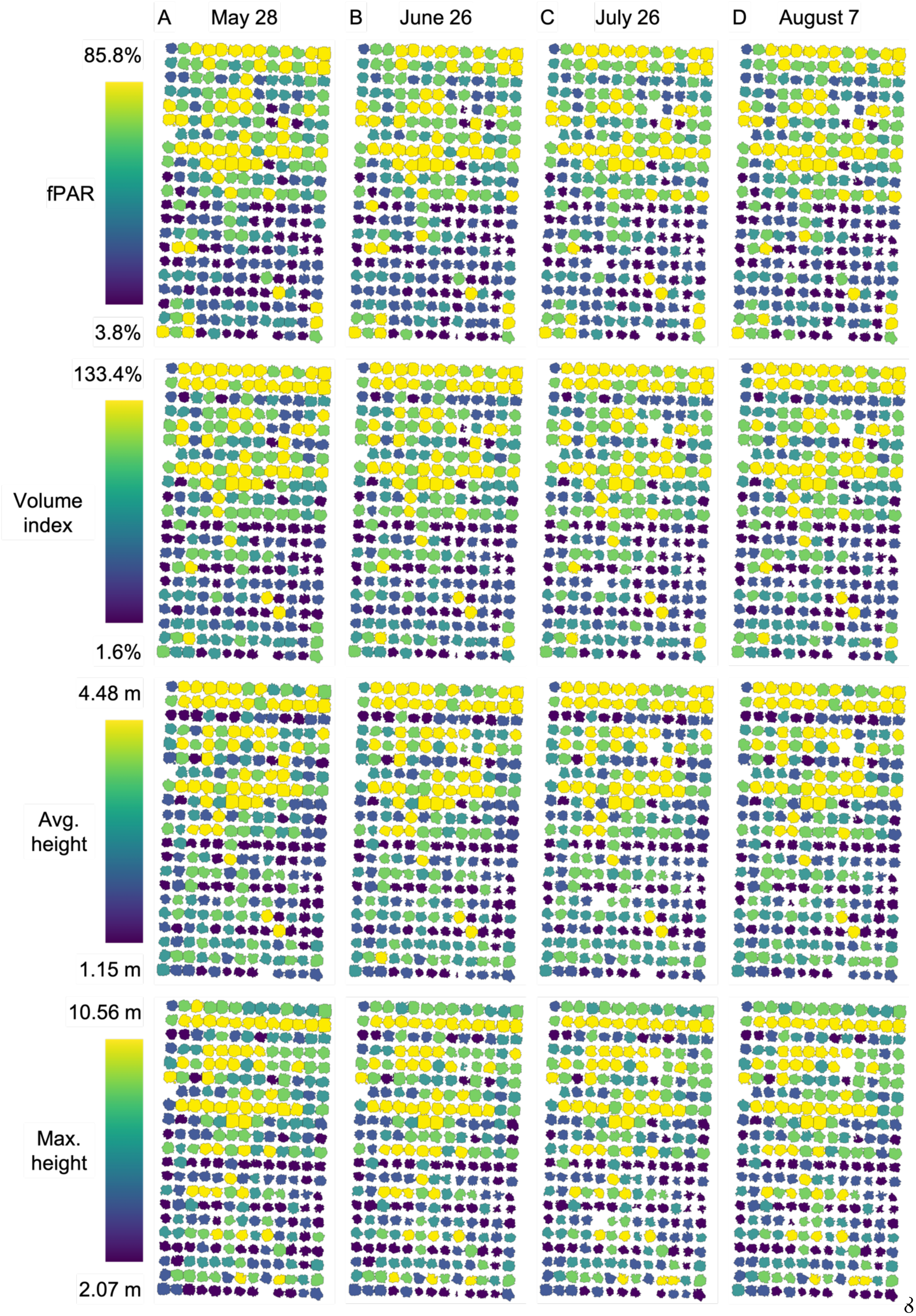
A
May 28
B
June 26
C
July 26
D
August 7
85.8%
fPAR
3.8%
133.4%
Volume index
1.6%
4.48 m
Avg. height
1.15 m
10.56 m
Max. height
2.07 m

Supplementary Figure 5. The visualization of all four extracted canopy profile features derived from digital surface models (DSMs), including fractional photosynthetically active radiation (fPAR), canopy volume index, average canopy height, and maximum canopy height, at the per-tree level for experimental almond orchard 3 (May 28 (**A**), June 26 (**B**), July 26 (**C**), and August 7 (**D**) in 2019).
